# Genomic variation in baboons from central Mozambique unveils complex evolutionary relationships with other *Papio* species

**DOI:** 10.1186/s12862-022-01999-7

**Published:** 2022-04-11

**Authors:** Cindy Santander, Ludovica Molinaro, Giacomo Mutti, Felipe I. Martínez, Jacinto Mathe, Maria Joana Ferreira da Silva, Matteo Caldon, Gonzalo Oteo-Garcia, Vera Aldeias, Will Archer, Marion Bamford, Dora Biro, René Bobe, David R. Braun, Philippa Hammond, Tina Lüdecke, Maria José Pinto, Luis Meira Paulo, Marc Stalmans, Frederico Tátá Regala, Francesco Bertolini, Ida Moltke, Alessandro Raveane, Luca Pagani, Susana Carvalho, Cristian Capelli

**Affiliations:** 1 Department of Biology, University of Copenhagen, Copenhagen, Denmark; 2 Department of Zoology, University of Oxford, Oxford, UK; 3 Estonian Biocentre, Institute of Genomics, University of Tartu, Tartu, Estonia; 4 Department of Biosciences, University of Milan, Milan, Italy; 5 Department of Chemistry, Life Sciences and Environmental Sustainability, University of Parma, Parma, Italy; 6 Escuela de Antropología, Facultad de Ciencias Sociales, Pontificia Universidad Católica de Chile, Santiago, Chile; 7 School of Anthropology, University of Oxford, Oxford, UK; 8 BIOPOLIS Program in Genomics, Biodiversity and Land Planning, CIBIO, Campus de Vairão, Vairão, Portugal; 9 CIBIO, Centro de Investigação em Biodiversidade e Recursos Genéticos, InBIO Laboratório Associado, Campus de Vairão, Universidade do Porto, Vairão, Portugal; 10 ONE - Organisms and Environment Group, School of Biosciences, Cardiff University, Sir Martin Evans Building, Cardiff, UK; 11 Interdisciplinary Center for Archaeology and Evolution of Human Behavior (ICArEHB), Universidade do Algarve, Faro, Portugal; 12 Department of Archaeology, National Museum, Bloemfontein, South Africa; 13 Evolutionary Studies Institute, University of the Witwatersrand, Johannesburg, South Africa; 14 Gorongosa National Park, Sofala, Mozambique; 15 Center for the Advanced Study of Human Paleobiology, George Washington University, Washington, USA; 16 Emmy Noether Group for Hominin Meat Consumption, Max Planck Institute for Chemistry, Mainz, Germany; 17 AESDA – Associação de Estudos Subterrâneos e Defesa do Ambiente, Lisbon, Portugal; 18 Department of Scientific Services, Gorongosa National Park, Chitengo, Sofala Province Mozambique; 19 Laboratory of Hematology-Oncology, European Institute of Oncology IRCCS, Milan, Italy; 20 Department of Biology, University of Padua, Padua, Italy

**Keywords:** Evolutionary genetics, Primate genomics, *Papio*, Population genomics

## Abstract

**Background:**

Gorongosa National Park in Mozambique hosts a large population of baboons, numbering over 200 troops. Gorongosa baboons have been tentatively identified as part of *Papio ursinus* on the basis of previous limited morphological analysis and a handful of mitochondrial DNA sequences. However, a recent morphological and morphometric analysis of Gorongosa baboons pinpointed the occurrence of several traits intermediate between *P. ursinus* and *P. cynocephalus*, leaving open the possibility of past and/or ongoing gene flow in the baboon population of Gorongosa National Park. In order to investigate the evolutionary history of baboons in Gorongosa, we generated high and low coverage whole genome sequence data of Gorongosa baboons and compared it to available *Papio* genomes.

**Results:**

We confirmed that *P. ursinus* is the species closest to Gorongosa baboons. However, the Gorongosa baboon genomes share more derived alleles with *P. cynocephalus* than *P. ursinus* does, but no recent gene flow between *P. ursinus* and *P. cynocephalus* was detected when available *Papio* genomes were analyzed. Our results, based on the analysis of autosomal, mitochondrial and Y chromosome data, suggest complex, possibly male-biased, gene flow between Gorongosa baboons and *P. cynocephalus*, hinting to direct or indirect contributions from baboons belonging to the “northern” *Papio* clade, and signal the presence of population structure within *P. ursinus*.

**Conclusions:**

The analysis of genome data generated from baboon samples collected in central Mozambique highlighted a complex set of evolutionary relationships with other baboons. Our results provided new insights in the population dynamics that have shaped baboon diversity.

**Supplementary Information:**

The online version contains supplementary material available at 10.1186/s12862-022-01999-7.

## Background

**Fig. 1:**
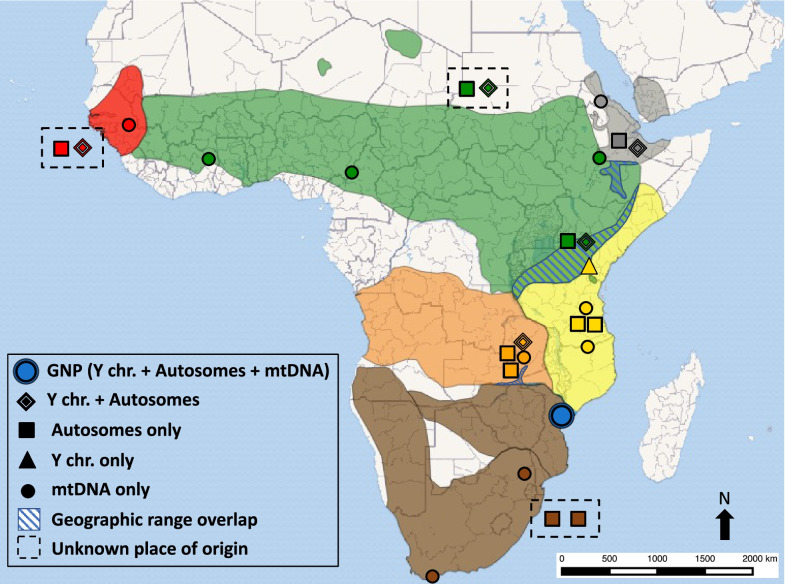
Distribution of *Papio* species and location of samples analyzed. Ychr.: Y chromosome sequence data; mtDNA: mitochondrial DNA genome. Shapes refer to the inset legend. Samples whose original place of origin is unknown are enclosed in a dashed box. Color scheme of *Papio* species distribution is as follows: *P. papio* = red*, P. anubis* = green*, P. kindae* = orange*, P. cynocephalus* = yellow*, P. ursinus* = brown*, and GNP*= blue. (Information on sample provenance collected from Additional file 2: Table S3 in Rogers et al. [11], Table 1 in Zinner et al. [10] and Appendix S1 in Wall et al. [15])

Baboons (genus *Papio*) are the primate genus beside humans with the largest distribution across Africa [1]. Over the last two million years they came to occupy a variety of different habitats, from the gallery forests of western Africa all the way to the rocky Cape Peninsula in southern Africa, extending their presence also outside Africa into the southwestern part of the Arabian Peninsula [2, 3]. Members of this genus are characterized by relatively extensive morphological and behavioral variation, such that their taxonomic classification is debated [2–4]. Most of the disagreement stems from what species concept is used, as their parapatric distribution, ecological flexibility and interfertility challenge many of the taxonomic criteria used for species classification [5–9]. A consensus has been recently reached supporting six extant baboon species (morphotypes, sensu Jolly et al. [9]) which are phylogenetically grouped in two geographic clades, one northern including *Papio papio, P. anubis* and *P. hamadryas,* and one southern comprising *P. cynocephalus*, *P. ursinus,* and *P. kindae* [4, 7, 10–13] (Fig. 1). *Papio* genetic diversity appears to be further characterized by a complex history of ancient and more recent gene flow, to include a deep admixture event for the origin of *P. kindae*, the contribution from a now extinct lineage to *P. papio*, and historical contacts between *P. cynocephalus* and *P. anubis* [11].

Admixture among different baboon species has been reported numerous times and the existence of several hybrid zones across the sub-saharan African distribution of the genus has been suggested [10, 13–16]. MtDNA data and microsatellite analysis have suggested contacts between populations of *P. hamadryas*, *P. anubis* and *P. cynocephalus* in Eastern Africa, and between *P. cynocephalus* and *P. ursinus*, as well as and *P. ursinus* and *P. kindae* in Southern Africa [4, 13, 14]. More recently, genome data provided some indication of the spatial extent of *P. anubis* introgression in *P. cynocephalus* populations in a contact zone in Eastern Africa [15] and hybridization between *P. kindae* and *P. ursinus* in the Kafue River Valley of Zambia [17].

Interestingly, despite female philopatry in several *Papio* species and the observation in the field of male-biased admixture dynamics [3, 4, 15], little molecular work has been conducted on Y chromosome variation in *Papio*. Phylogenetic studies including *Papio* species have surveyed only a few thousands bp of Y chromosome sequence, while population-based studies have screened a handful of Y chromosome specific SNPs and STRs [18–21]. As such, virtually no large Y chromosome datasets have been systematically investigated for population and evolutionary studies. The analysis of Y chromosome sequences hundreds of kilo-bases (kb) in length retrieved from genomic data is therefore expected to provide not only a high resolution male-specific insight of *Papio* phylogeny but also to give access to hundreds of Y chromosome markers useful for population studies addressing sex-biased interactions [4], as shown for great apes including humans [22].

Overall, gene-exchange appears to be common among neighboring groups of baboons; however, the extent and the dynamics of these events are yet to be fully appreciated within *Papio* and the occurrence of gene flow should be tested in a more systematic way across other potential areas of contact between different species [10]. Understanding these processes at the genome level is of particular relevance to reconstruct the demographic and evolutionary dynamics that have shaped the diversity of behavior, social structure and phenotype seen in baboons, and has significant implications for interpreting the evolutionary history of other species, extant and extinct, including hominins [2, 3].

Two species of baboons, *P. cynocephalus* and *P. ursinus,* are present in Mozambique in south-eastern Africa (Fig. 1), whose distribution has been inferred by a combination of molecular and morphological data [4, 12, 23]. The Zambezi River, a major hydrological feature which runs east to west through central Mozambique, has been suggested as the natural barrier dividing the regions of occupation of the two species [10, 24]. Baboons south of the Zambezi have been assigned to the subspecies *P. ursinus griseipes* (i.e. grayfoot chacma baboons) one of the three morphotypes identified within *P. ursinus* [25]. The Zambezi has been suggested as a biogeographical barrier for other species as well, for example Blue wildebeest (*Connochaetes taurinus*) and sable antelope (*Hippotragus niger*), but it is not yet fully understood how permeable to migration this barrier has been over time [26–28].

As part of a project assessing primate adaptations and evolution in the southern African Rift System [29, 30] we recently investigated the morphological variation of baboons located approximately 100 km south of the Zambezi River, within Gorongosa National Park (Fig. 1) [23]. The park hosts a large population of baboons, which comprises more than 200 groups [31] which have been previously assigned to *P. ursinus griseipes*, in line with the clustering of Gorongosa mitochondrial DNA with lineages sampled in the northern area of *P. ursinus* distribution [10, 25]. However, cranial morphometric variation placed Gorongosa baboons in between *P. ursinus* and *P. cynocephalus*, closer to *P. ursinus,* but with several phenotypes observed in Gorongosa described as a mosaic of features of the two species [23].

Given the potential for baboons in Gorongosa National Park having experienced interspecies admixture, we posit to investigate their evolutionary history, including testing for evidence of gene flow, using a genomic approach. We generated low and high coverage genome sequences of two baboons from Gorongosa National Park, and compared them to available genomes from other baboon species revealing a complex evolutionary relationship between baboons in Gorongosa, and other members of the genus *Papio*. We complemented the whole genome analysis with the investigation of uniparental markers, exploring whole mitogenome data and Y chromosome sequences several hundreds of thousand nucleotides in length, providing female and male specific insights into the phylogenetic relationships of Gorongosa baboons within the genus *Papio*.

## Results

### Whole genome sequence of baboon samples from Gorongosa National Park, Mozambique

We generated the whole genome sequence of a baboon individual sampled in Gorongosa National Park, in central Mozambique (sample GNP). The median coverage across the genome was 36.7X. The X chromosome was reported having a coverage of 18X, approximately half of the coverage reported for chromosome 20 (35X), consistent with the ratios observed and corresponding information on sex (Additional file 2: Table S1) for baboon samples included in Rogers et al. [11]. This led us to conclude that the sample was originally collected from a male individual. Out of the more than 36 million variants identified across the whole genomic dataset analyzed in this study (GNP and [11]), 1,488,924 variants were found to be private to the GNP baboon. We filtered variants to remove singletons, retaining a final set of 36,538,410 biallelic polymorphisms.

We additionally generated whole genome sequence data from a blood sample also collected from a Gorongosa baboon (sample BB1). The quality of the DNA was such that the mean coverage across the genome was only 0.03X. The reads were mapped to the Panu_3.0 reference genome through BWA-MEM (v. 0.7.17-r1188).

### Mitochondrial phylogenetics

**Fig. 2:**
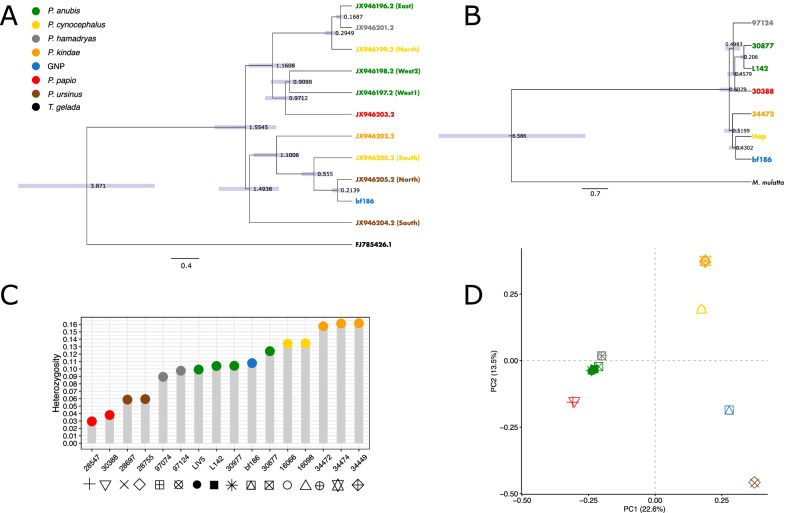
GNP baboon genomic variation and *Papio* diversity. **A** Phylogeny of *Papio* mitogenomes using *Theropithecus gelada* as an outgroup; codes refer to the specimen identifiers used in Zinner et al. [10]. GNP: baboon sample collected in Gorongosa National Park (code: bf146); colors indicate the different species listed in the legend. All nodes have a posterior probability of 1. Divergence times are reported (confidence intervals shown as gray boxes; values in Tables S2). **B** Y chromosome *Papio* phylogeny based on six concatenated genes (see main text); codes as in Additional file 2: Table S1; Hap refers to a *P. cynocephalus* male sample from Wall et al. [15]; colors refer to legend in panel **A**. All nodes have a posterior probability of 1. Divergence times are reported (confidence intervals shown as gray boxes; values in Tables S3). *Macaca mulatta* was used as an outgroup. **C** Heterozygosity estimates across *Papio*; colors as legend in panel **A**; sample codes as reported in Additional file 2: Table S1. **D** Principal component analysis (PCA) of *Papio* autosomal genomic data; codes and colors as in panel legend in panel **A**

As part of the genome sequencing, we also retrieved the whole mitogenome of the GNP baboon. GNP mtDNA sequence matched the few hundred base pairs previously analyzed in samples collected in Gorongosa [13, 25]. To fully explore the mitochondrial variation, we assembled a reference dataset of *Papio* mitogenomes and reconstructed the phylogenetic relationship of our sample and other baboon mitogenomes [10] (Figs. 1; 2A). *P. ursinus* mitogenomes are located in two distinct parts of the phylogenetic tree, one comprising samples from the southern part of the *P. ursinus* range and one clustering the samples further north [10, 21]. The GNP mitogenome clustered with the northern *P. ursinus* samples, in a clade also including *P. kindae* and southern *P. cynocephalus*. Moreover, we dated the different nodes in the tree, obtaining dates in line with previous results [10]. The Time to the Most Recent Common Ancestor (TMRCA) for the GNP/Northern *P. ursinus* mtDNA clade was 0.21 Mya (confidence interval: 0.14–0.29) (Additional file 2: Table S3). The TMRCA of the next node, extended to include the mtDNA of a southern *P. cynocephalus* sample, was dated to 0.55 Mya (0.38–0.73), while the clade including all *P. ursinus* mtDNA lineages dated to 1.49 Mya (1.06–1.94) (Additional file 2: Table S3). We also generated a tree using Zinner et al. [10] dataset and all of the mitogenomes recovered from whole genome sequence data (GNP and [11]) (Additional file 1: Fig. S2). The tree showed a topology identical to the one presented in Fig. 2A; TMRCAs were broadly similar, the CIs being larger but in line with the estimates generated by Mathieson et al. [21] using the same whole genome data from Rogers et al. [11] to extract mitogenomes.

### Y chromosome analysis

We extended our genomic analysis to include sequences from six Y chromosome genes: *SRY, DDX3Y, KDM5D, ZFY, UTY, USP9Y*. Approximately 456 kb were recovered for each of the seven baboon males available to us: the GNP sample, the five males present in the Baboon Genome Diversity Panel [11] (Additional file 2: Table S1; Fig. 1; see Methods section) and a previously published genome of a male *P. cynocephalus* individual [15]. *P. ursinus* male whole genome sequence data was not available. We aligned the *Papio* sequences including *M. mulatta* Y chromosome data as an outgroup and retrieved a concatenated section of Y chromosome 425 kb in length. A total of 2,098 variable positions were found across *Papio* Y chromosomes, of which 267 variants were detected only on the GNP Y chromosome (Additional file 2: Table S4). We used the retrieved sequences to generate a phylogenetic tree (Fig. 2B), the first exploring Y chromosome variation over hundreds of thousands of nucleotides across *Papio* species. The topology of the tree supported a separation of Y chromosome baboon lineages in two clades, one comprising only northern baboon species and the other only southern baboon individuals. A similar separation was reported when genomic and mtDNA data were analyzed [10, 11] (Fig. 2A). The closest Y chromosome lineage for the GNP sample belongs to *P. cynocephalus* from Amboseli National Park in southern Kenya [15]. Interestingly, the mtDNA lineages of Amboseli *P. cynocephalus* have been shown to cluster within the northern clade of the mitochondrial phylogeny [13]. The divergence time for the GNP/*P. cynocephalus* clade was dated to 0.43 Mya (C.I. 0.26–0.63 Mya), and the TMRCA of the whole southern baboons’ clade (*P. cynocephalus, P. kindae* and GNP) was dated to 0.52 Mya (0.32–0.75 Mya). The TMRCA for the whole of the Y chromosome *Papio* lineages here included was dated to 0.61 Mya (0.38–0.87 Mya) (Additional file 2: Table S5).

### Population genomics of Gorongosa baboons

The GNP sample was compared to Rogers et al. [11] baboon genome data using the genomic variants called as described in Material and Methods. The heterozygosity for each genome was estimated as the fraction of the total number of variable sites being heterozygous in a given individual (Fig. 2C; Additional file 2: Table S1). Baboons sampled in captivity (*P. ursinus* and *P. papio*) were characterized by lower SNP heterozygosity than individuals collected in the field, as previously reported [11]. The estimate for the GNP sample at 0.12 is in the middle of the heterozygosity range for other baboons (0.03–0.16), slightly lower than values reported for *P. cynocephalus* (0.13) but twice as much of that observed in *P. ursinus* (0.06) (Fig. 2C). Principal Components Analysis (PCA) was implemented to visualize the relationship of the GNP baboon with other *Papio* species (Fig. 2D). PC1 separated baboons along the same North–South division observed in the mtDNA (with the exception of *P. cynocephalus* being paraphyletic) and Y-chromosome analyses and previously reported for genomic data [11]. PC2 further separated southern baboons in two groups: *P. kindae*/*P. cynocephalus* and *P. ursinus*/GNP, the GNP sample located in between *P. cynocephalus* and *P. ursinus*, closer to the latter. The low coverage sample BB1 was closest to GNP when projected on the PCA, the two separated to a certain extent along PC1. A similar behavior was observed when GNP was downsampled to a coverage comparable to that of BB1 (i.e. GNP_ds) and projected on the PCA, suggesting that the departure of BB1 from GNP is affected mostly by the degree of genome coverage more than the occurrence of significant differences between the two Gorongosa samples (Additional file 1: Fig. S1A).

The genus *Papio* is characterized by a rich history of admixture between species [11, 15, 17] and Gorongosa baboons display a mosaic of phenotypic features [23]. We therefore tested if the genomic variation of the GNP sample could be modeled as the result of an admixture event involving different baboon species. As a control, we ran each of the analyses performed on the GNP sample on sample 30877, an individual known to be admixed between *P. anubis* and *P. cynocephalus* [11] (Supplementary Information).

**Fig. 3:**
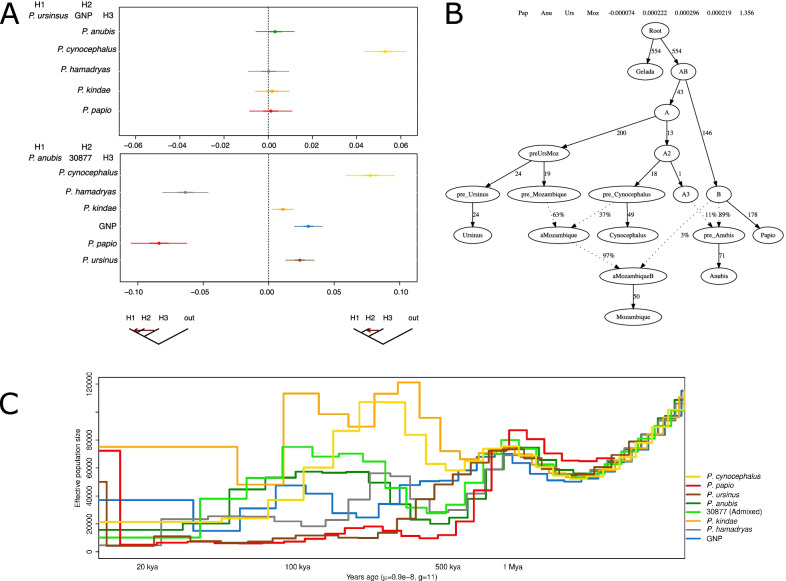
Genomic history of GNP and other baboons. **A** Patterns of shared alleles (*D*-statistics results). Upper panel: *P. ursinus* and GNP comparisons; lower panel: *P. anubis* and *P. anubis* 30877 comparisons. H1 and H2 refer to the two populations being compared to the test population, H3. Bars show the extension of three standard deviations; thicker parts refer to a single standard deviation. Colors as in Fig. 2. **B** Reconstruction of the genetic relationships between different *Papio* species with the addition of admixture events using qpGraph. GNP and all *Papio* species, except for *P. kindae* and *P. hamadryas*, are included. The *f*_4_ statistics (Z = 1.356) with poorest correlation, reported at the top, do not reject the topology in the figure. The label “Mozambique” refers to sample GNP. **C** Changes in effective population sizes. The results of the PSMC analysis for GNP and one individual for each *Papio* species, including all sites. Full results in Additional file 1: Fig. S5

We used the *f*_3_ statistics in the form of *f*_3_ (GNP; X, Y), where X and Y represented different baboon species [11]. None of the tested pairs provided significant (Z ≤ − 3) *f*_3_ results for GNP (Additional file 2: Table S6). We then investigated to what extent GNP and *P. ursinus* were similarly related to other *Papio* species. We implemented the *D*-statistics in the form of *D*(*T. gelada*, X, GNP, *P. ursinus*), X representing individuals belonging to other *Papio* species. GNP resulted significantly enriched in *P. cynocephalus* alleles relative to *P. ursinus* (Z = 5.31 and Z = 5.37, when compared to the two *P. ursinus* samples, 28755 and 28697, respectively) (Fig. 3A). The Gorongosa sample BB1 was not significantly different from GNP when the two were tested in the form *D*(*T. gelada*, *P. cynocephalus*, GNP, BB1) (Additional file 1: Fig. S1B). Notably, both showed significant differences when compared to *P. ursinus* for the number of shared alleles with *P. cynocephalus* (Additional file 1: Fig. S1). Testing GNP *vs.* other species did not generate significant departures from zero; however, when *P. anubis* baboons were included in the analysis, the *D* value diverged from zero more than others, even if not significantly. We quantified the amount of *P. cynocephalu*s ancestry present in the GNP genome using the *f*_4_ ratio alpha in the form *f*_4_(gelada, cynocephalus, GNP, ursinus)/*f*_4_(gelada, cynocephalus; cynocephalus, ursinus). The estimated value of alpha was 8% (Additional file 2: Table S7). On the contrary, no significant contribution of *P. cynocephalus* was detected in GNP when the *f*_4_ ratio was calculated using X chromosome SNPs (Additional file 2: Table S7). We also attempted local ancestry deconvolution of the *P. ursinus*/*P. cynocephalus* components in GNP using ELAI but failed to retrieve any *P. cynocephalus* contribution (Additional file 2: Table S8; Supplementary Information). This outcome is in line with the non-significant *f*_3_ admixture tests but contrasts with the *f*_4_ ratio results. Comparatively, known admixed baboon 30877 gave significant values for both *f*_3_ and *D-*statistics when set-up similarly to GNP tests (i.e. *P. anubis* in place of *P. ursinus*), in addition to an *f*_4_ ratio alpha that yielded approximately a *P. cynocephalus* contribution of 11% (Additional file 2: Table S7). Local ancestry analysis conducted using *P. anubis* individuals as recipients and *P. cynocephalus*/other *P. anubis* as donors consistently identified *P. cynocephalus* ancestry within 30877, but a smaller fraction was detected when 30877 was the only *P. anubis* sample included as target (11% and 7% respectively; Additional file 2: Table S8, Supplementary Information). *P. cynocephalus* contribution to the *P. anubis* individuals here analyzed was dated 10–20 generations ago using Local Ancestry and linkage-based approaches (Additional file 1: Fig. S7, Additional file 2: Table S9; Supplementary Information). No substantial differences were observed when a recombination map was included in the analysis (Additional file 2: Table S8; Additional file 1: Fig. S8). Interestingly, the heterozygosity of 30877 decreased when SNPs included in regions assigned as having a *P. cynocephalus* origin were excluded, reaching values in line with other *P. anubis* samples (Additional file 2: Table S2; Supplementary Information).

To gather additional insights on the evolutionary relationships of GNP with other baboons we explored different tree-topologies using qpGraph (Fig. 3B; Additional file 1: Fig. S3). We initially tested the tree proposed by Rogers et al. [11]. Major features of this tree are a subdivision in northern and southern baboons, *P. kindae* modeled as the result of the admixture between northern and southern baboons, *P. papio* identified as the recipient of gene-flow from an unknown *Papio* lineage and *T. gelada* assigned as an outgroup to *Papio* variation. This proposed topology was not supported, in particular the use of *T. gelada* as a full outgroup to *Papio* (Additional file 1: Fig. S3). We therefore started from a simpler topology and subsequently added different lineages, including admixture events (Supplementary Information). The addition of GNP to the tree required the inclusion of contributions from sources related to both *P. cynocephalus* and a proto northern baboon population (~ 37% and ~ 3%, respectively). The inclusion of only one of the two contributions resulted in unsupported topologies (data not shown). QpGraph analysis supported the inclusion of *P. anubis* only when an admixture event involving 11% contribution from *P. cynocephalus* was added (Fig. 3B). Contacts between *P. cynocephalus* and GNP/*P. anubis* were also supported by Treemix analysis (Additional file 1: Fig. S4). The most supported trees in Treemix included either one or five migration edges between different branches. All trees reported a migration edge between the *P. ursinus* lineage and *P. kindae*. The additional edges connected *T. gelada* with *P. hamadryas* and *P. papio,* and *P. cynocephalus* with *P. anubis* and GNP (Additional file 1: Fig. S4).

Finally, we attempted to reconstruct the changes in population size across time of the Gorongosa population. We examined the distribution of coalescent times across the genome of the GNP sample and all the other genomes here included [11] using the PSMC software. A selection of the PSMC runs (one individual per species) is included in Fig. 3C, together with the results of the analysis of the GNP baboon (full results in Additional file 1: Fig. S5). These PSMC runs replicate results already published in addition to providing a direct comparison for the GNP sample which was analyzed here for the first time [11]. The GNP genome showed fluctuations in population sizes between 500 and 100 kya. At 50 kya, GNP baboon population size was similar to other *Papio* populations in the wild, but higher than the one reported by the two species sampled in captivity, *P. ursinus* and *P. papio*. Interestingly, population sizes for GNP and *P. ursinus* started to diverge at the time when they began to decrease, around 500 kya (Fig. 3C). Notably admixture events can lead to fluctuations in effective population size, sometimes denoted in PSMC curves as large humps or, in more complex scenarios, as increasing and decreasing humps over time [32, 33]. Fluctuations in size between 500 and 100 kya are present in both the ancestral populations of GNP and 30877, markedly so when compared to *P. ursinus* and *P. anubis,* respectively (Fig. 3C; Additional file 1: Fig. S5). Nevertheless, ancient population structure cannot be ruled out for the GNP baboon when also taking into account the discordant results obtained from the admixture tests performed [34, 35].

The PSMC analysis of GNP mapped to Panubis1.0 was consistent with results observed using genomic data mapped to Panu_3.0. (Additional file 1: Fig. S6).

## Discussion

### Gorongosa National Park baboons in the context of Papio variation

The recent publication of whole genome sequences of samples representative of the six different baboon species has provided the context for investigating the variation recorded in other baboon populations [11]. We used this resource to investigate the genomic variation recovered from two baboons in Gorongosa National Park, Mozambique (GNP and BB1, high and low coverage, respectively). Southeast Africa is a region that has been only marginally investigated so far in relation to the local baboon genetic variation [4, 13, 17, 25]. Autosomal and mtDNA data were consistent in suggesting a closer affinity for Gorongosa baboons to *P. ursinus* (Fig. 2; Additional file 1: Fig. S1). The extensive dataset available for *Papio* mitogenomes provided further resolution within *P. ursinus*, and confirmed previous results placing GNP mitochondrial lineage closer to baboon from the northern part of *P. ursinus* range (Fig. 2A; Zinner et al. [10]). The TMRCA for GNP and the closest *P. ursinus* mtDNA lineage was dated to around 200 kya, while the earliest coalescent event with *P. cynocephalus* was more than 500 kya. We also extended our phylogenetic analysis of uniparental markers to the Y chromosome, analyzing more than 400 kb of the non-recombining part of the Y chromosome. No *P. ursinus* Y chromosomes were available for comparison and the closest Y chromosome lineage to GNP belonged to a *P. cynocephalus* individual from Amboseli National Park, southern Kenya [10, 13–16], with a TMRCA dated to 0.38 Mya (Fig. 2B). Y chromosome and mtDNA results are broadly in line in identifying Gorongosa baboons as belonging to “the southern baboons’ lineage” described in Zinner et al. [13], with similar dates for the mtDNA divergence time with *P. cynocephalus*. However, the mtDNA dates for the overall antiquity of southern baboons, with or without *P. ursinus* lineages from the south (1.10 Mya and 1.49 Mya respectively; Additional file 2: Table S2), were much older than the estimate obtained for the Y chromosome for the GNP/*P. kindae*/*P. cynocephalus* clade (0.52 Mya; Fig. 2B). Such discrepancies could be related to our limited survey of Y chromosome variation across baboons, in particular the absence of Y chromosome types belonging to *P. ursinus* sampled further south. But we note that this is also the case when the overall antiquity of the *Papio* clade was considered (0.61 Mya and 1.55 Mya, for the Y chromosome and the mtDNA, respectively) and limited sampling alone might not be enough to explain these results. Male-biased dispersal and polygynous mating patterns have been proposed as ways to explain Y chromosome TMRCAs significantly younger than mtDNA ones observed in gorillas (genus *Gorilla*) and orangutans (genus *Pongo*) [22]. A refined Y chromosome phylogeny generated by including a wider and more informative dataset will make it possible to investigate the impact of such behaviors on the genetic variation of *Papio*.

### The genomic history of Gorongosa baboons

Gorongosa baboons have been historically defined as belonging *to P. u. griseipes,* because the geographical location of Gorongosa falls within its range of distribution (i.e. IUCN, [36]). However, the taxonomic assignment of baboons from the Lower and Middle Zambezi region (Malawi, eastern Zambia, and north-central Mozambique) are not yet fully understood [9]. Recent morphological/craniometric analyses reported a mix of *ursinus/cynocephalus* features in this population [23].

Despite *P. ursinu*s being the *Papio* species closer to Gorongosa baboons, the genomes of the Gorongosa specimens were different from *P. ursinus* samples. GNP and *P. ursinus* had well differentiated mtDNA lineages (Fig. 2A), presented different degrees of variation (Fig. 2C), were separated along PC2 (Fig. 2D; Additional file 1: Fig. S1), and were characterized by different demographic histories (Fig. 3C). The two groups also differed in their affinity with other *Papio* lineages, as the GNP genome resulted closer to *P. cynocephalus* than *P. ursinus* (Fig. 3A) and showed a potential contribution from an additional source (Fig. 3B). BB1 results based on low coverage data were consistent with those of GNP (Additional file 1: Fig. S1). Contrary to *P. anubis* 30877, *f*_3_ and ELAI results did not support a simple recent admixture scenario for the increased affinity of GNP to *P. cynocephalus*. Combined, the *f*_3_, *f*_4_ ratio, ELAI and *D-*statistics suggest a complex relationship between GNP and *P. cynocephalus*. Male-mediated introgression of *P. ursinus* into *P. cynocephalus* (the “nuclear swamping” scenario) has been suggested to explain the relatedness between the mtDNA lineages present in northern *P. ursinus* and southern *P. cynocephalus* [37], a hypothesis compatible with our genome-wide results. Interestingly, the GNP X chromosome, differently from the rest of the genome, does not show a closer affinity to *P. cynocephalus*, suggesting that contacts might have been male mediated. However, if indeed male-biased gene flow was responsible for the observed autosomal/X imbalance, introgression would have been operating, from *P. cynocephalus* to *P. ursinus*, opposite to what was previously proposed. Male hybrid sterility is an alternative explanation for rapid depletion of introgressed X chromosomes. Both have been proposed to explain similar patterns observed in modern humans in relation to Neanderthal introgression [38], but infertility has not been reported across the different *Papio* hybrid zones identified so far [2]. One of the topologies supported by qpGraph (Fig. 3B; Additional file 1: Fig. S3) suggests the occurrence of subdivision within *P. ursinus* and contacts between the GNP lineage and a lineage related to *P. cynocephalus*. An origin of *P. cynocephalu*s from a population of proto *P. ursinus* closer to Gorongosa baboons, and subsequent gene flow among *P. ursinus* populations, including GNP complements our findings. A similar scenario, labeled as ancient population structure, has been reported to inflate *D* results and was initially suggested to explain the increase in shared variants between non-African populations and Neanderthals [39]. Both scenarios (gene flow and ancient population structure) assume the presence of deep population subdivision within *P. ursinus,* which is supported by previous surveys of *P. ursinus* mtDNA variation [25]*.* The deep TMRCA of GNP/*P. ursinus* mtDNA clade and the PSMC results suggest a separation (complete or partial) between the tested *P. ursinus* and GNP genome dating to hundreds of thousands of years ago. We also note here that *P. cynocephalus* from the north (as the ones available here) might not represent an appropriate proxy of the true source population and the non-significant *f*_3_ results might have been driven by the occurrence of population structure in *P. cynocephalus*, which is suggested by mtDNA data [13] (Fig. 2A), and supported by the identification of multiple morphotypes in yellow baboons [9]. Similarly, drift operating on the three populations since the event of admixture might have affected the *f*_3_ results [40].

The indication of additional contributions to GNP related to proto northern baboons is more difficult to interpret given our current limited understanding of baboon genomic diversity. We also note that the evidence suggesting this contact is based on only one set of analyses (qpGraph). One possible explanation might be that northern baboon variants entered Gorongosa via interactions with Kinda baboons. *P. kindae* have been suggested as originating from an ancient admixture event between northern and southern baboons. However, neither the *f*_3_ results nor the *D*-statistics support a direct GNP/*P. kindae* admixture (Additional file 2: Table S6, Fig. 3A). Alternatively, northern baboon ancestry might have entered the Gorongosa population via *P. ursinus* populations mixed with *P. kindae*. Notably, *kindae/ursinus* admixture has been detected in Zambia [4, 17]. Unexpected variation might have also originated from more exotic sources, including different Papionini. Intergenera interactions have been highlighted for *P. cynocephalus/R. kipunji* using mtDNA data, and *Papio/Theropithecus* interactions have been reported in captivity and directly observed in the wild, and suggested on the basis of transposable elements detected in both genera [41–44], the latter compatible with our qpGraph and Treemix results (Additional file 1: Figs. S3, S4). The characterization of these occurrences and the relevance for Gorongosa baboons are definitely worth additional investigation, which will be enabled by the addition of other Gorongosa genomes in the future.

Finally, we note that the lack of significant reduction in genetic variation reported for the GNP genome (Fig. 2C) is of interest in the context of the decrease in size of local populations reported for large mammals after the Mozambican Civil War from 1976 to 1992 [31]. In combination with the steady increase in the number of troops since 1994 and an effective population size similar to other *Papio* species in their more recent evolutionary history (Fig. 3C), this makes the morphological and genetic variation of Gorongosa baboons a valuable resource to investigate the selection-based dynamics that have shaped local diversity. Notably, the curves for captive samples of *P. ursinus* and *P. papio* are indicative of a long-term reduction in population size, dating back to hundreds of thousands of years. This opens up the possibility that the low degree of heterozygosity displayed by these individuals might not simply be the result of their captive status but instead reflect a more general reduction in diversity in the populations in the wild from which they descend. It is relevant here to note that mtDNA analysis suggested an overall reduction in variation for *P. papio* [45, 46].

### Implications for our understanding of the evolutionary history of the genus *Homo*

The results gathered from the analysis of Y chromosome, mitochondrial and genome data have highlighted the presence of complex dynamics across *Papio* populations in southern Africa. The timeline of these events spans the last few hundreds of thousands of years, a time when phenotypically differentiated *Homo* populations were present in the same area. It is tempting to speculate on the significance of the parallels between *Homo* and *Papio* in the region: the occurrence of morphologically different *Homo* populations in southern Africa more than 230 kya (e.g. *H. naledi*, Florisbad and Kabwe) [47, 48] and the genomic differentiation reported for GNP and other *P. ursinus* individuals. More data is necessary to explore the full extent of the genomic variation present across *P. ursinus* subpopulations. Nevertheless, investigating how morphology and genomics map onto each other across *P. ursinus* morphotypes and providing a chronological context for their relationships is expected to help develop an interpretative framework for the phylogenetic significance of the phenotypic diversity observed in *Homo*. In this context the observation of shared polymorphisms between GNP/BB1 and *P. cynocephalus* is particularly enlightening as it suggests that relationships extending further north might have been also present among *Homo* groups, much in line with the African metapopulation model that has been proposed for the origin of our own species [49]. The further integration of climatic records is expected to highlight the role played by ecological changes in shaping events of drift, gene flow, isolation and extinction in the region in both *Papio* and *Homo* [50].

## Conclusions

Gorongosa National Park in southeastern Africa lies within the region occupied by *P. ursinus griseipes* (grayfoot baboons), which is one of three morphotypes identified within the *P. ursinus* morphological variation [9]. Our results provided evidence for increased allele sharing between baboons in Gorongosa and *P. cynocephalus*, not related to recent gene flow. A mosaic of *P. ursinus/P. cynocephalus* features have been reported in Gorongosa baboons, but to which extent *P. cynocephalus* genetic variants affected their morphology remains unclear [23]. Similarly, if and how much the occurrence of *P. cynocephalus* variants extends into other grayfoot baboon populations and into other *P. ursinus* morphotypes is still unknown. Additional Gorongosa genomes collected from morphologically characterized individuals and the sampling across different populations of *P. ursinus* are expected to clarify the dynamics that shaped the phenotypic variation and the evolutionary relationships of populations and species of baboons in southern Africa. Finally, extensive genomic survey of *Papio* diversity, including Y chromosome, will provide additional insights on the extent of within-species population structure and interspecies sex-biased gene-exchange, contributing to the development of an interpretive framework for the phylogenetic significance of the phenotypic diversity observed in other genera in the region, including *Homo* [2].

## Materials and methods

### Sample collection and DNA extraction

DNA to generate high coverage whole genome sequence was obtained from a muscle tissue sample collected post-mortem as a result of an infanticide that took place in Gorongosa, Mozambique, in 2017. The infanticide occurred in the early afternoon in Chitengo Safari Camp (coordinates lat: − 18.97727; long: 34.35140). A sample of muscle tissue was collected ca. four hours after the death of the animal. As in other baboons of similar age in Gorongosa [23], the infant’s pelage was black, but no other morphological details were recorded, as the body was left in the open after sample collection to continue monitoring the spatial dynamics of the members of the troop around the body. By evening, the carcass was taken by a genet (*Genetta genetta*).

We additionally collected a blood sample in 2019 from a sedated female adult individual belonging to a troop different from the one roaming in and near Chitengo to which the infant (Sample GNP) belonged (coordinates of the collected sample: lat − 18.9051644; long 34.3760742; ca. 8 km north of Chitengo). The animal was sedated to be collared as part of the Paleo Primate Project Gorongosa (PPPG), to track the movement of the troop.

DNA was initially extracted from tissue and blood at the Molecular Genetics laboratory in Chitengo using DNeasy Blood and Tissue kit (Qiagen), with minor improvements from the manufacturer's protocol (e.g. overnight digestion of tissue sample using proteinase K). The DNA extracts were further purified using RNAse at the Research Centre in Biodiversity and Genetic Resources (CIBIO, Vairão, Portugal). Extracted DNA was quantified with a *Qubit* fluorometer using the Qubit dsDNA BR Assay kit (Invitrogen) following manufacturer's instructions. Genome sequencing was performed by Edinburgh Genomics (Edinburgh, UK). The tissue sample from the infanticide is here referred to as GNP (code: bf186). The blood sample coagulated after collection and as a result only a small amount of DNA could be recovered; this sample is here referred to as BB1.

### DNA sequencing and genome mapping

Genome sequencing was performed by Edinburgh Genomics (Edinburgh, UK). The GNP sample was sequenced to an average depth of 36.8× using Illumina HiSeq X platform (150 bp paired-end reads). The integrity of the sequenced sample yielded a total of 121.4 Gb of data, with 98.8% of reads mapped to the Panu_3.0 assembly. We aligned our Illumina reads using BWA-MEM 0.7.17-r1188 [51] to baboon reference Panu_3.0 assembly and generated a BAM file which was used for further downstream analyses. Duplicate reads were marked and removed using Picard MarkDuplicates version 2.8.1 [52]. A total of 100 Mb sequence data of the BB1 genomic DNA was generated using a MiSeq instrument, which resulted in a 0.03X coverage once mapped to Panu_3.0. Given the limited amount of data, BB1 was only used for Principal Components Analysis and *D*-statistics, to provide further insights in the genomic variation present in Gorongosa baboons, as described below.

After we started to analyze the GNP sample, an additional baboon reference genome became available (Panubis1.0 [53]). Structural differences between the Panu_3.0 and Panubis1.0 assemblies have been highlighted. We explored the impact a different assembly might have on our results by re-mapping the GNP genomic data to Panubis1.0 and using it to run PSMC analysis on GNP only (see below). We want to note here, that a systematic evaluation of how two assemblies might differ when used for population genomics investigations is out of the scope of this manuscript. We therefore limited this comparison to a subset of analyses that are potentially affected by differences in the assemblage of contiguous genomic sections, such as PSMC.

### Sex determination

The injuries suffered by the GNP individual and the very young age prevented a morphological identification of the sex of the animal. We therefore attempted to sex it by investigating the genome coverage across chromosomes. As males comprised only one X chromosome, the average X chromosome coverage should be approximately half of that for the autosomes, which are present in pairs. The sex of the individual was established by comparing the average coverage reported for sex chromosome X and the coverage obtained for autosomal chromosome 20. We validated this approach after Rogers et al. [11]; we also included the three samples for whom the sex was not provided in the 2019 paper (codes: 28547, 30877, 30977). Additionally, the sex of the GNP baboon was confirmed using a molecular sex determination protocol that simultaneously amplifies via Polymerase Chain Reaction (PCR) fragments of the amelogenin X gene and the SRY Y-linked gene [54] (data not shown).

Sample BB1 was taken from a female baboon while she was sedated to place a radio-collar on her.

### Mitochondrial DNA analysis

We first downloaded the complete mitochondrial genomes originally published by Zinner et al. [10]. We then mapped the reads of the GNP individual, as well as the reads of each baboon from the Baboon Genome Diversity Panel [11], to the mitochondrial reference for *Papio anubis* found in the papAnu2 assembly (NC_020006.2). To obtain a consensus mitogenome from whole genome sequence data we used ANGSD [55]. A fasta file was created for each sample, where consensus sequences were called per individual using the highest effective depth. A minimum base quality of 30 and a minimum of 10 reads were used to consider a base for the consensus.

NGS consensus mitogenomes and Zinner et al. [10] mitogenomes were concatenated and aligned using the multiple sequence aligner, MUSCLE 3.8.31 [56], an algorithm best suited for nucleotide datasets. The resulting alignment of 16,569 bp was then further filtered using GBlocks 0.91b [57] to remove any indels and gaps unsuitable for phylogenetic analyses. The mtDNA alignment, with both whole genome sequence consensus mitogenomes and Zinner et al. [10] mitogenomes, was 16,402 bp in length. We used jModelTest2 [58] and IQ-TREE [59] to determine the best substitution model for our alignment based on the corrected Akaike inference criterion (AICc) and Bayesian inference criterion (BIC). Both AICc and BIC gave TrN + I + G as the best substitution model when using jModelTest2 and similarly, IQ-TREE gave TN + F + R3 as the best fit (i.e., TrN using a FreeRates model instead of a gamma distribution). We produced the best Maximum Likelihood (ML) tree with support values from 10,000 bootstraps. Similarly, we also produced an mtDNA alignment using only our GNP mitogenome and Zinner et al. [10] mitogenomes. For this alignment jModelTest2 and IQ-TREE both recommended GTR as the best substitution model.

Divergence dates for the mitogenomes were estimated using BEAST 2.6.3 [60]. A relaxed lognormal clock model was chosen to model lineage variation and birth–death process prior branching rates. Using the jModelTest2 recommendation we used a TrN + I + G site model for the dataset including Rogers et al. [11] mitogenomes while for the dataset using only Zinner et al. [10] mitogenomes and our GNP mitogenome we used a GTR site model. We provided a fossil-based calibration point for the *Theropithecus*-*Papio* node at 4 Mya (CI: 0.5 Mya) based on previous work [10]. Four replicates were performed for 25 million generations, tree and parameter sampling taking place every 1000 generations. We used Tracer 1.7.1v [61] to check whether each replicate, with a 10% burn-in, resulted in convergence of all parameters across generations. These sampling distributions were then combined using 25% burn-in with LogCombiner. A consensus tree was produced using TreeAnnotator where node heights were summarized to reflect posterior median node heights. Lastly, the consensus tree was visualized using FigTree v1.4.4 (http://tree.bio.ed.ac.uk/software/figtree). Two trees were produced: one including all the mitogenome sequences recovered from whole genome sequences (GNP and Rogers et al. [11]) plus the Zinner et al. [10] dataset, and one with only the Zinner et al. [10] dataset and the GNP mitogenome here assembled.

### Y chromosome analysis

The Baboon Genome Diversity Panel analyzed in Rogers et al. [11] included data for four males, one each from *P. anubis, P. papio, P. hamadryas* and *P. kindae*. We identified an additional male sample in the Rogers et al. [11] dataset by comparing the coverage of the X chromosome and chromosome 20 (sample 30877, *P. anubis*; Additional file 2: Table S1). To complement this set, we further included the genomic data of a *P. cynocephalus* male presented in Wall et al. [15], together with the genomic sequence of the male GNP baboon we generated, and the *M. mulatta* (NC_027914.1) Y chromosome reference sequence. We recovered data from six Y-specific single-copy genes (*SRY, DDX3Y, KDM5D, ZFY, UTY, USP9Y*) selected as described in the Supplementary Information. Preliminary phylogenetic trees were built using the ML algorithm and 100 bootstrap replicates. The best substitution model was evaluated with the “Find best DNA/Protein Models” function available in MEGAX [62]. The best substitution model, both in terms of BIC and AICc, was the GTR + G + I. The reconstruction of the divergence tree, based upon the best substitution model above identified, and molecular dating were performed with BEAST v1.10.4 [63]. The model chosen was an uncorrelated lognormal relaxed clock model with a Yule speciation model. The starting tree was built with the Unweighted Pair Group Method with Arithmetic Mean (UPGMA). One calibration point was applied as normal prior with a mean of 7 Mya, and a standard deviation of 1 Mya to the node containing all Papionini species [64]. The Markov chain Monte Carlo was run for 100 million generations sampling every 1000 generations. The log file resulting from the analysis was imported in Tracer v.1.7.1 [61] with a burn-in of 30% to assess convergence of all parameters by examination of ESS values. Finally, the consensus tree was produced using TreeAnnotator and visualized with FigTree v1.4.4 (http://tree.bio.ed.ac.uk/software/figtree).

### Genome comparisons

#### Variant calling

Following GATK best practices, we called variants for the autosomal chromosomes using GATK version 4.1.8.1 [65]. Indels were realigned using IndelRealigner and followed by HaplotypeCaller which was used to generate gVCFs for our sample. Initially, we performed joint genotype calling with 16 previously published baboons representing six species of *Papio* (nine individuals collected in the wild and seven from captive colonies) [11]. Joint calling was done as reported by Rogers et al. [11]. Filtered GATK variants were used in all the genome-based analyses, excluding the PSMC analysis where variants were determined using individual-based genotype calling with bcftools [66]. PSMC requires a consensus sequence score (FQ) alongside the consensus sequences for each individual; a calculation only provided by bcftools at this time. For consistency, we also replicated the genome-based analyses (PCA, *D*-statistics, heterozygosity) using these individual-based calls, the results agreeing with the ones based on the joint-calling (data not shown). All variants were filtered using the following hard-filters (SNPs: “QD < 2.0 || FS > 60.0 || MQ < 40.0 || MQRankSum <  − 12.5 || ReadPosRankSum <  − 8.0”; Indels: “QD < 2.0 || FS > 200.0 || ReadPosRankSum <  − 20.0”). We also filtered to exclude indels and multiple nucleotide polymorphisms providing a total of 56,030,625 SNPs (GATK). Only sites with bi-alellic calls were kept. Sites with a minor allele frequency (MAF) below 0.05 were removed. After filtering, a final set of 36,538,410 autosomal SNPs was used for further statistical analyses. Similarly, variants were called for the X chromosome where, after filtering, a set of 1,413,976 SNPs was obtained. Variant calling for BB1 was done using GATK v4.2.4.1 *HaplotypeCaller*, which generated a set of 3,716 SNPs in the final gVCF.

#### Heterozygosity

Using PLINK [67], we estimated heterozygosity as the proportion of heterozygous loci per-individual within each respective variant dataset (joint-called and individual-based calling). For comparison with previously reported results [11] we have also estimated the heterozygosity as the number of polymorphic sites over the total length of the ungapped autosomal scaffolds (Chr. 1–20) (Additional file 2: Table S2).

#### Principal components analysis (PCA)

In order to determine where the GNP sample fits within the population structure of baboons, we performed PCA on the 16 *Papio* individuals using PLINK [67] on both joint-called (GATK) and individual-based called (bcftools) variants.

We performed principal component analysis of low coverage data using the smartpca function implemented in EIGENSOFT [68] software 8.0.0. Specifically, we projected sample BB1, GNP and *P. ursinus* 28697 downsampled genotypes onto the PCA inferred from the 16 *Papio* individuals and GNP. The downsampling was done through Picard v.2.26.4 *DownsampleSam,* performing 10 replicates for GNP and *P. ursinus* 28697. The options -P 0.0003 and -R were used.

#### TreeMix

To better understand the topology and gene flow between the *Papio* species, we used TreeMix 1.13 [69] to determine the population tree using our GATK called variants and using PLINK to calculate the allele count per population. We ran TreeMix with migration edges that ranged from 0 to 10. For each migration edge, we performed 100 independent runs with bootstrap replicates using 500 SNPs per block. To evaluate the optimal number of migration edges in our population tree we used OptM [70]. We observed that with a single migration edge, more than 99.8% of the variance observed could be explained. Based on ∆*m*, the second most optimal number of migration edges was 5. The first time we observe any migration edge going into our GNP sample is at 4. For each population tree presented here we plotted the TreeMix replicate with the highest global likelihood after a convergence where the top 5 out of every 100 runs had similar likelihoods (± 50).

#### PSMC

We inferred the effective population size for each baboon across time using PSMC. The recommended procedure to run PSMC requires generating diploid sequences per individual with a FQ score using bcftools [66]. Sites with a coverage below 10 and greater than three times the genome-wide coverage per individual were excluded as well as sites called heterozygous with less than three reads of support. PSMC was applied with default settings. We used a mutation rate of 0.9 × 10^–8^ and a generation time of 11 years as estimated by Rogers et al. [11]. Additionally, we performed the same procedure excluding repetitive regions as indicated by RepeatMasker [71]. The analysis was repeated for the GNP sample when mapped to Panubis1.0.

#### *D*-statistics

To test for differences in allele sharing between populations (H_1_, H_2_) relative to different baboon species (H_3_) we performed *D-*statistics (ABBA-BABA) using POPSTATS [72]. The *Z*-scores were calculated using the weighted jack-knife procedure where a 5 Mb block weighted by the number of loci was used to estimate the standard error [73, 74].

Given the much lower coverage of BB1, we also performed *D-*statistics (ABBA-BABA) using ANGSD [55]. We used the option -doAbbababa 1, an approach that samples a random base at each position of a BAM file to estimate the counts of ABBA and BABA sites between H1, H2, and H3 and is therefore particularly suitable for low coverage samples. Z-scores were calculated based on a jackknife procedure [74] for blocks of data that were 5 Mb in size, performed through the R-script available on the ANGSD github website (https://github.com/ANGSD/angsd/tree/master/R). We initially tested if BB1 and GNP shared a similar number of alleles with the two available *P. cynocephalus* samples (16,066 and 16,098; Additional file 2: Table S1). We then independently compared BB1 and GNP to each of the two available *P. ursinus* samples (28697 and 28755, Additional file 2: Table S1) to test for differences in the number of shared alleles with the two *P. cynocephalus* genomes.

#### *f*_3_ statistics

The presence of admixture events was tested using the *f*_3_-admixture statistics via qp3Pop as implemented in AdmixTools version 6.0 [40]. We performed the test in the form *f*_3_(Pop1,Pop2,Target), where the target was either the GNP genome or one *P. anubis* individual, and Pop1 and Pop2 were a combination of available populations.

#### *f*_4_ ratio (autosomal and X chromosome data)

We performed the f_4_-statistics using the AdmixTools qpDstat program, with the setting f4mode: YES [40]. *f*_4_-statistics measures the amount of shared drift in a defined four population tree topology [40, 73]. Significant deviations of the f_4_-statistics from 0 indicate that the tree topology does not fit the data and suggest higher shared genetic drift between clusters than expected. It is possible to leverage on these properties of the *f*_4_-statistics to estimate the global proportions of an ancestry in an admixed population, through the *f*_4_-ratio test [73].

We use the format *f*_4_(A, Target, B_1_, gelada) and *f*_4_(A, B_2_, B_1_, gelada) to infer the numerator and the denominator of the ratio, respectively. In turn, the quotient indicates the proportion of gene flow from B_1_ to Target. We maintained the same *f*_4_-statistics format when focusing on the X chromosome, considering only one individual per population to ensure matching numbers of X chromosomes across samples. Given the inclusion of both males and females for the X chromosome analysis, for the male samples we opted to perform the *f*_4_-statistic in a hybrid form, thus computing the statistic between individuals of different sex.

#### qpGraph

To visualize the relationships and admixture proportions between the studied samples we used the qpGraph package included in AdmixTools [40]. Given a defined topology, where the target populations are set as terminal leaves and internal nodes are pseudo-populations, the tool computes *f*_2_, *f*_3_, and *f*_4_-statistics to evaluate the amount of genetic drift between the target groups. QpGraph returns the defined topology where branch lengths and admixture proportions are estimated from the computed F-statistics, along with the worst *f*_4_-statistic Z-score. In this study, we consider the defined topology supported when the worst *f*_4_-statistics Z-score is |Z|< 3. We set the option outgroup: NULL for all tree-like models, except when we replicated the topology presented in [11], where we set *T. gelada* as the formal outgroup of the graph.

#### Local ancestry (ELAI)

Local Ancestry Inferences (LAI) identify the ancestral fragments in an admixed genome and assign them to the putative sources of the admixture event. After statistically phasing the samples using SHAPEIT [75], we performed LAI with ELAI, a tool based on a two layers Hidden Markov Model able to detect haplotype structure between the source populations as well as within each source. This feature allows ELAI to handle delicate scenarios where the ancestry segments are short, and where there is a grade of haplotype variability within the source samples [76]. ELAI is shown to be able to deconvolute target individuals even when only a few source samples are available [77], therefore proving to be an ideal tool for this study. We ran ELAI [76] by averaging 10 independent runs, each of them characterized by 20 steps of inference optimization, as recommended by ELAI developers. We indicated 15 admixture generations (-mg), two upper clusters (-C 2) and eight lower clusters (-c 8). Downstream analyses were performed after removing all SNPs that could not be assigned to one ancestry with a mean local ancestry dosage less than 0.8. We perform the analysis of three different sets of test genomes: (i) the GNP sample, considering as putative source populations *P. cynocephalus* and *P. ursinus*; (ii) the 30,877 sample from *P. anubis,* as a mixture of *P. cynocephalus and P. papio*; (iii) all four individuals of the *P. anubis* population, selecting as putative sources *P. cynocephalus* and *P. papio*.

Statistical phasing of samples for LAI and dating ancestry switches was performed with SHAPEIT [75] in two ways. One phasing was performed using a uniform recombination rate of 1 × 10^–8^ per base pair per generation [11] while another was done using the recombination map generated from pedigreed captive baboons [78]. The recombination map produced using a block penalty value of 5 was used as those were the rates focused on in the Robinson et al. [78] study. Recombination rates for the Panu_3.0 assembly from the Panu_2.0 assembly were generated using the liftOver chain provided by Robinson et al. Furthermore, regions which were found to be erroneously assembled in Panu_3.0 [53] were masked for both aforementioned analyzes.

#### Dating ancestry switches

We harvest ELAI results to calculate the number of generations occurring since the admixture event on the basis of the number of ancestry switches occurring along chromosomes, following the equation reported in Johnson et al. [79]. The equation considers two types of recombination events to count the number of ancestry switches: within the haploid genome (consisting in a genome having a single copy of each chromosome) and between two haplotypes of opposite ancestry. The expected number of recombination events in a haploid genome is computed as 0.01 × *TL*, with *T* being the number of generations and *L* the total genome length. The recombination events occurring between two haplotypes of opposite ancestry are indicated as 2 × *a*(1 − *a*), where *a* stands for the genome-wide ancestry proportion.

Thus, the expected number of ancestry switches in a diploid genome is:

$$B = \left( {2 \times 2 \times 0.01} \right) \times TL \times a\left( {1 - a} \right)$$

We computed the admixture event *T* from the observed *B* (ancestry switches) after [79]. The equation was applied on haploid genomes (after phasing), therefore resulting in:

$$T \, = \, B/(2 \times 0.01) \times L \times a(1 - a)$$

#### ALDER and MALDER

We compute weighted LD statistics to date the admixture events by running both ALDER [80] and MALDER [81] on the *P. anubis* dataset, considering different bin sizes (0.0005, 0.0001 and 0.001). All other parameters were set as default. The availability of a single genome prevented the implementation of these analyses on the GNP sample.

## Supplementary Information

**Additional file 1.** Distribution, location, and heterozygosity of samples (**Figure S1**). mtDNA phylogenetics (**Figure S2**). Y chromosome data & phylogenetics. qpGraph (**Figure S3**). TreeMix (**Figure S4**). PSMC (**Figures S5–S6**). Characterizing *P. cynocephalus* ancestry in *P. anubis* (**Figures S7–S8**).

**Additional file 2. Table S1.** Samples information, including genomic coverage for Chromosome X and Chromosome 20. **Table S2.** Impact of P. cynocephalus ancestry on *P. anubis* 30877 heterozygosity. **Table S3.** TMRCAs and C.I. for mtDNA tree in figure 1. **Table S4.** Number of differences between Y chromosome sequences analysed in figure 1b. **Table S5.** TMRCAs and C.I. for Y chromosome tree in figure 1b. **Table S6.** f3 results. **Table s7.** f4 ratio alpha estimates. **Table S8.** Local Ancestry estimations (Global Proportions obtained with cutoff of 0.8). **Table S9.** ALDER and MALDER results (All combinations of *Papio* species were tested as PopA/PopB; only significant results are reported here).

## Data Availability

The genomic data of samples GNP and BB1, supporting the conclusions of this article, are available under the ENA accession study number PRJEB52124. The GitHub repository for this paper can be found here: https://github.com/santaci/GNPbaboons.

## Abbreviations

TMRCA: Time to most recent common ancestor
mtDNA: Mitochondrial DNA
NGS: Next generation sequencing
CI: Confidence interval
PCA: Principal component analysis
LD: Linkage disequilibrium
LAI: Local ancestry inference
Mya: Million years ago
kya: Thousand years ago
UPGMA: Unweighted pair group method with arithmetic mean
WGS: Whole genome sequencing
PSMC: Pairwise Sequentially Markovian Coalescent
MAF: Minor allele frequency
