# Supplementary material for: Genomic variation in baboons from central Mozambique unveils complex evolutionary relationships with other Papio species
Source: BMC Ecol Evol. 2022 Apr 11;22:44. doi: 10.1186/s12862-022-01999-7 (PMC8996594; doi:10.1186/s12862-022-01999-7)
Supplement: Supplementary file 1 — Additional file 1. Distribution, location, and heterozygosity of samples (Figure S1). mtDNA phylogenetics (Figure S2). Y chromosome data & phylogenetics. qpGraph (Figure S3). TreeMix (Figure S4). PSMC (Figures S5–S6). Characterizing P. cynocephalus ancestry in P. anubis (Figures S7–S8). [file 12862_2022_1999_MOESM1_ESM.pdf]

Supplemental Information for:

Genomic variation in baboons from central Mozambique unveils complex evolutionary relationships with other *Papio* species.

Cindy Santander, Ludovica Molinaro, Giacomo Mutti, Felipe I. Martínez, Jacinto Mathe, Maria Joana Ferreira da Silva, Matteo Caldon, Gonzalo Oteo-Garcia, Vera Aldeias, William Archer, Marion Bamford, Dora Biro, René Bobe, David R. Braun, Philippa Hammond, Tina Lüdecke, Maria Pinto, Luis Meira Paulo, Marc Stalmans, Frederico Tátá, Francesco Bertolini, Ida Moltke, Alessandro Raveane, Luca Pagani, Susana Carvalho, Cristian Capelli

Table of Contents:

**Distribution, location, and heterozygosity of samples**	Page 2
**mtDNA phylogenetics**	Page 3
**Y chromosome data & phylogenetics**	Page 4
**qpGraph**	Page 6
**TreeMix**	Page 9
**PSMC**	Page 10
**Characterizing *P. cynocephalus* ancestry in *P. anubis***	Page 11

Distribution, location, and heterozygosity of samples

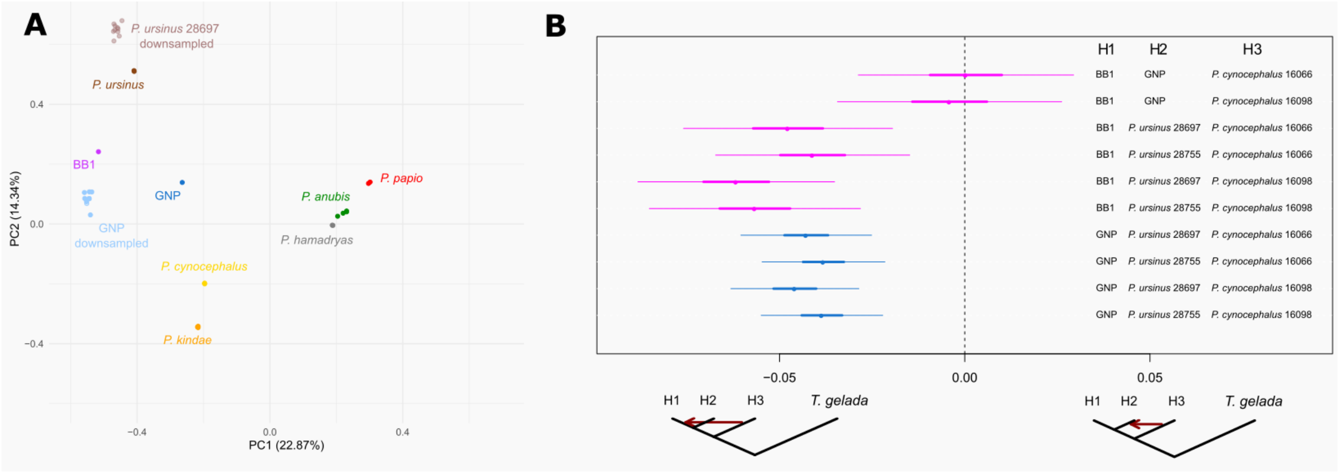

Figure S1. BB1 in PCA and *D*-statistics analyses.

a. Principal component analysis (PCA) of BB1, 10 replicates of GNP downsampled and 10 replicates of *P. ursinus* 28697 downsampled projected onto the components inferred from autosomal genomic data of *Papio sp.*
b. *D*-statistics results (ANGSD). In purple, BB1 compared to GNP, *P. ursinus* 28697 and *P. ursinus* 28755. In blue, GNP compared to *P. ursinus* 28697 and *P. ursinus* 28755. H1 and H2 refer to the two individuals being compared to the test individuals, H3 (*P. cynocephalus* 16066 and *P. cynocephalus* 16098). Bars show the extension of three standard deviations; thicker parts refer to a single standard deviation.

Table S1. *Please see Supplementary Tables spreadsheet, Table S1.* Samples information, including X chromosome and chromosome 20 coverage and sequencing runs used for retrieving Y chromosome data.

Table S2. *Please see Supplementary Tables spreadsheet, Table S2.* Heterozygosity estimates (overall and based on 30877 local ancestry results).

Mitochondrial phylogenetics

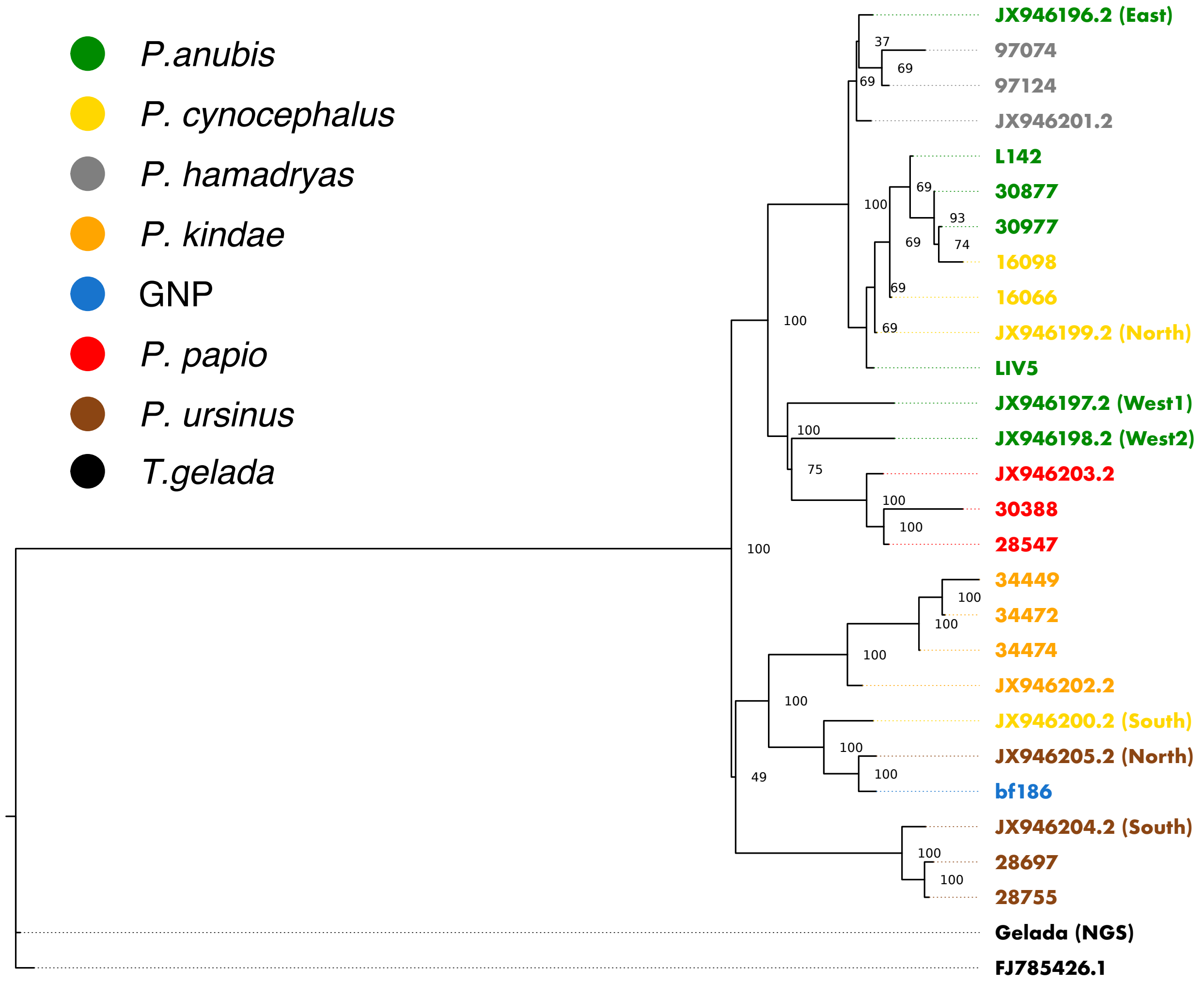

Figure S2. Maximum Likelihood phylogeny of *Papio* mitogenomes including mitogenomes recovered from genomes here analyzed (GNP and Rogers et al., [1]) and from Zinner et al., [2]. Codes as in [2] and Table S1.

Table S3. *Please see Supplementary Tables spreadsheet, Table S3.* TMRCAs and confidence intervals for the mtDNA phylogeny in main Figure 1a.

Y chromosome sequence data and phylogenetics

We downloaded the male genomes from [1] and one male sample (HAP) from Wall et al. [3] using the NCBI SRA Toolkit (https://github.com/ncbi/sra-tools/wiki). When multiple runs per sample were present, as the case for all the sequences present in [1], the runs to be downloaded were selected following three criteria: 1) runs generated using the same Illumina library; 2) HiSeq2000 as the platform used for sequencing; and 3) the most recent runs produced for each sample. A total of 16 runs for the five male samples in Rogers et al. [1] were retrieved (Table S1).

We used a Snakemake [4] singularity- conda- dependent pipeline to align, merge (if multiple runs per sample were present) and generate the consensus fasta sequence for the seven male WGS sequences (six from the literature and one newly generated). More in detail, we used bwa-mem2 algorithm [5] to align the genomic reads to the Y chromosome *P. anubis* reference (NC_044997.1) and including the Panu_3.0 assembly. In the case of multiple runs, we merged them with samtools [6]. Duplicates were marked with Picard 2.22.1 [7] and removed with samtools [6]. The consensus sequence for each sample was generated using ANGSD [8].

We focused our analysis of Y chromosome variation on a selected set of genes. To avoid downstream issues related to the mapping of genomic reads, we considered only Y chromosome-specific genes occurring in single copy and chose the 13 present in *Macaca mulatta* [9]. The mammalian Y chromosome is characterized by regions (strata, S) that started diverging from the X chromosome at different times in the past. S1, S2 and S3 have been suggested to have diverged from the X chromosome since more than 100 Ma, while the S4 and S5 regions emerged possibly only 40 Ma. To minimize the risk of X chromosome reads cross-mapping on the Y chromosome sequences, we decided to focus on the eight single-copy genes present in the S1-3 regions. Of these eight, S1 and S2 host only one gene each (*SRY* and *KDM5D*, respectively), while six genes are present in S3 (*ZFY, TMSB4Y, UTY, USP9Y, EIF1AY, DDX3Y*). Of these eight genes only six were

retained because they were annotated and identified for all the species in the analysis. FASTA gene sequences were extracted by blasting the *P. anubis* annotated SRY, DDX3Y, KDM5D, ZFY, UTY, USP9Y sequences on each of the newly generated consensus Y chromosome using BLASTn [10] exploiting the BLAST+ interface [11]. We built the sequence alignments for single genes using ClustalW as implemented in MEGAX [12] and then we concatenated with an in-house R script. The Multiple Sequence Alignment (MSA) was then filtered using Gblocks 0.91b [13] to remove poorly aligned positions and divergent regions. Further, all the positions missing from one or more chromosomes were excluded. The sequences of the genes recovered from the fasta Y chromosome reference sequence available for *M. mulatta* (NC_027914.1) were used as an outgroup.

Table S4. *Please see Supplementary Tables spreadsheet, Table S4.* Number of differences among Papio Y chromosome lineages analyzed in main Figure 1b.

Table S5. *Please see Supplementary Tables spreadsheet, Table S5.* TMRCAs and confidence intervals for the Y chromosome phylogeny in Figure 1b.

qpGraph analysis

We initially tested the tree proposed by [1], but the results were not supported, a major violation to the tree topology being the use of gelada as a full outgroup (Figure S3). However, such topology was not supported even allowing for gelada being no formal outgroup (Figure S3). Instead of moving backward from this topology by sequentially modifying different parts of it, we decided to start by evaluating the support for simpler topologies. We therefore began by including the two southern baboons suggested to be relevant for GNP (*P. ursinus*, *P. cynocephalus)* and a geographically well separated northern baboon species (*P. papio).* This simplified topology was supported by qpGraph without requiring any additional events of gene flow, with gelada not a formal outgroup (Figure S3). We then proceeded by adding GNP to the southern baboon part of the tree. In doing so we initially tested three alternative branching patterns: i) (GNP (*P. ursinus, P. cynocephalus*)); ii) (*P. cynocephalus* (GNP, *P. ursinus);* iii) (*P.ursinus* (*GNP, P. cynocephalus*). None of these trees was supported; however, results were not equivalent across topologies. The tree including the clade (*P. cynocephalus* (*P. ursinus*, GNP)) was the only one with a non-zero amount of shared drift for the internal branch (Figure S3). The zero shared drift between *P. cynocephalus* and GNP or *P. ursinus* also rejected the additional alternative scenario of a sudden split of these three groups (trifurcation). We then modified the tree with GNP and *P. ursinus* as sister groups by exploring different admixture scenarios. In order to be supported, the tree needed to include contributions to GNP from sources related to *P. cynocephalus* and *P. papio* (~37% and ~3%, respectively). The inclusion of only one of the two contributions resulted in topologies not supported (data not shown). In order to test the ability of qpGraph in recovering *Papio* evolutionary relationships in the presence of gene flow, we finalized this topology by adding *P. anubis*. QpGraph analysis supported the inclusion of *P. anubis* only when an admixture event involving 11% contribution from *P. cynocephalus* was also added in the tree (Figure 2b).

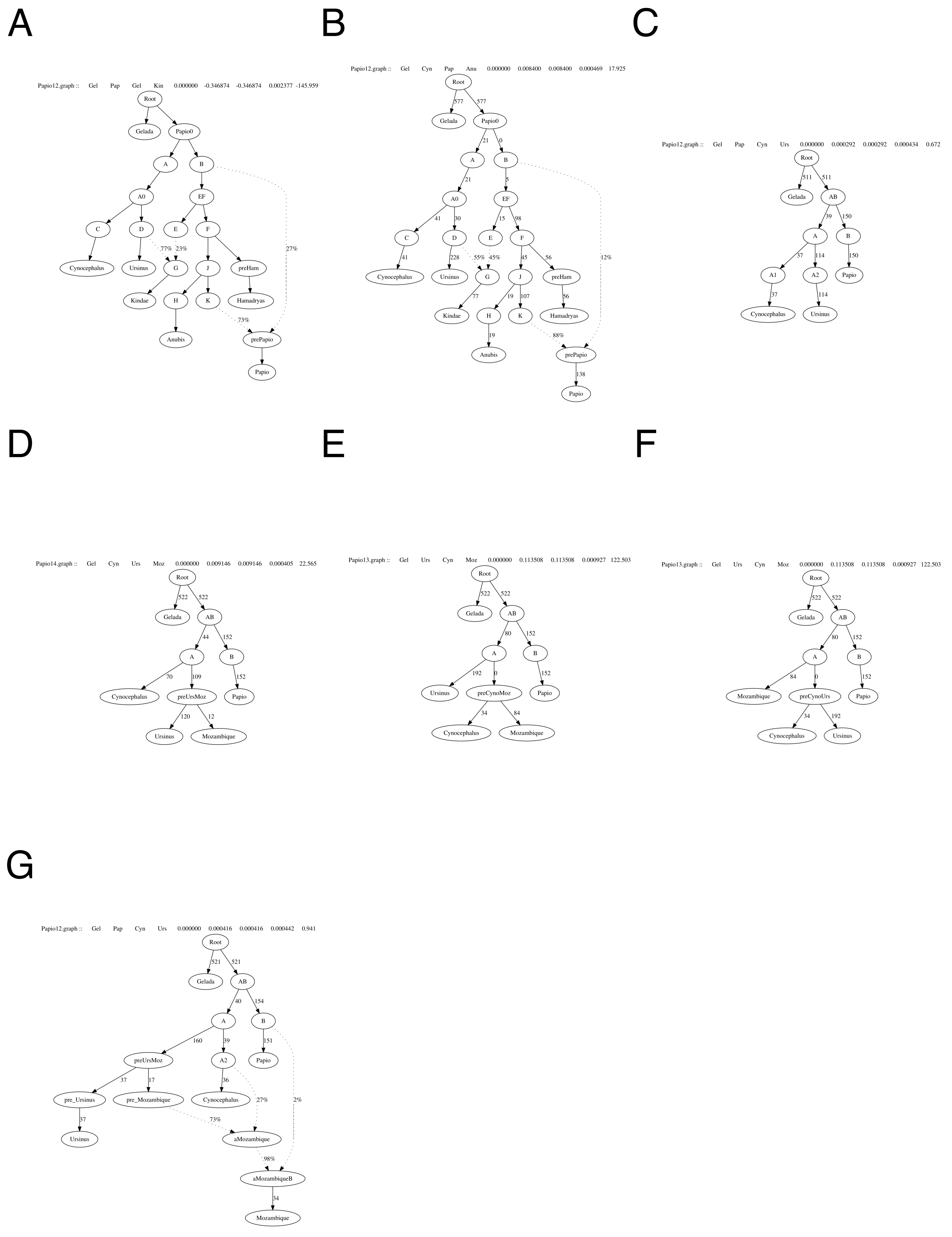

Figure S3. qpGraph results. The Z values of the worst f_4 statistics are reported at the top of each graph. (A) Phylogenetic tree based on results presented in [1]; *T.gelada* full outgroup. (B)

Phylogenetic tree based on results presented in [1]; *T.gelada* no full outgroup. (C) Phylogenetic tree including only *P. cynocephalus, P. ursinus, P. papio*. *T.gelada* no full outgroup. (D) Phylogenetic tree as in panel c, with the addition of GNP; tested topology as follows: (*P. papio*, (*P. cynocephalus, (P. ursinus, GNP))). T. gelada* no full outgroup. (E) Phylogenetic tree as in panel c, with the addition of GNP; tested topology as follows: (*P. papio,* (*P. ursinus (P. cynocephalus, GNP))). T.gelada* no full outgroup. (F) Phylogenetic tree as in panel c, with the addition of GNP; tested topology as follows: (*P. papio, (GNP, (P. cynocephalus, P. ursinus))). T.gelada* no full outgroup. (G) Phylogenetic tree as in panel c, including gene flow events for GNP. *T.gelada* no full outgroup

TreeMix

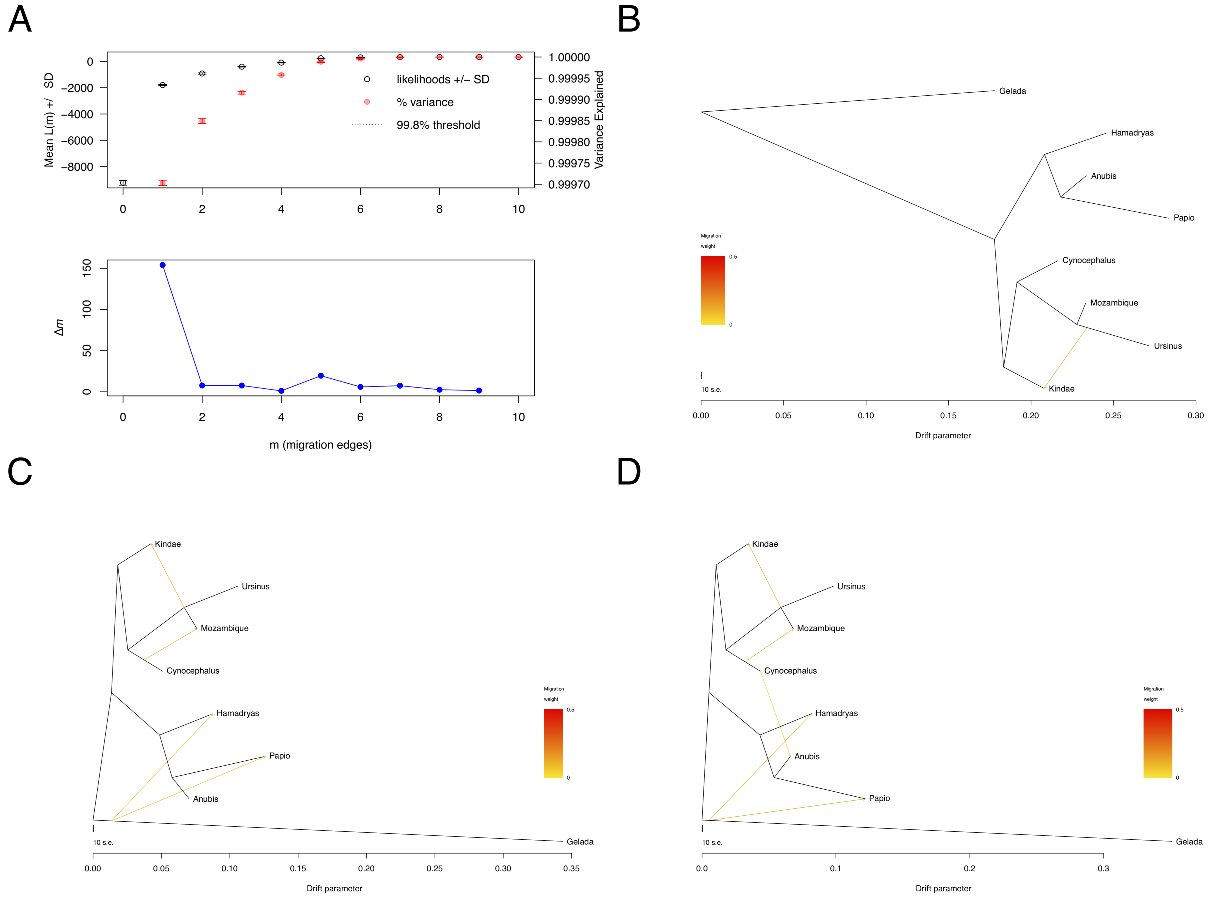

Figure S4. Treemix results. Treemix analyses using *T. gelada* as an outgroup. (A) OptM results for Treemix exploring m = 1-10, k = 500. Top: Variance explained by each migration edge added to the tree without priors. More than 99.8% of the variance can be explained with the one migration edge alone. Bottom: *ad hoc* statistic Δ*m* evaluating optimal number of migration edges on the tree. (B) Treemix graph with a single migration edge, the most optimal number of migration edges based on OptM. (C) Treemix graph where a migration edge is first observed going into GNP (from *P. cynocephalus* branch); m = 4. (D) Treemix graph with the second most optimal number of migration edges; m = 5.

PSMC

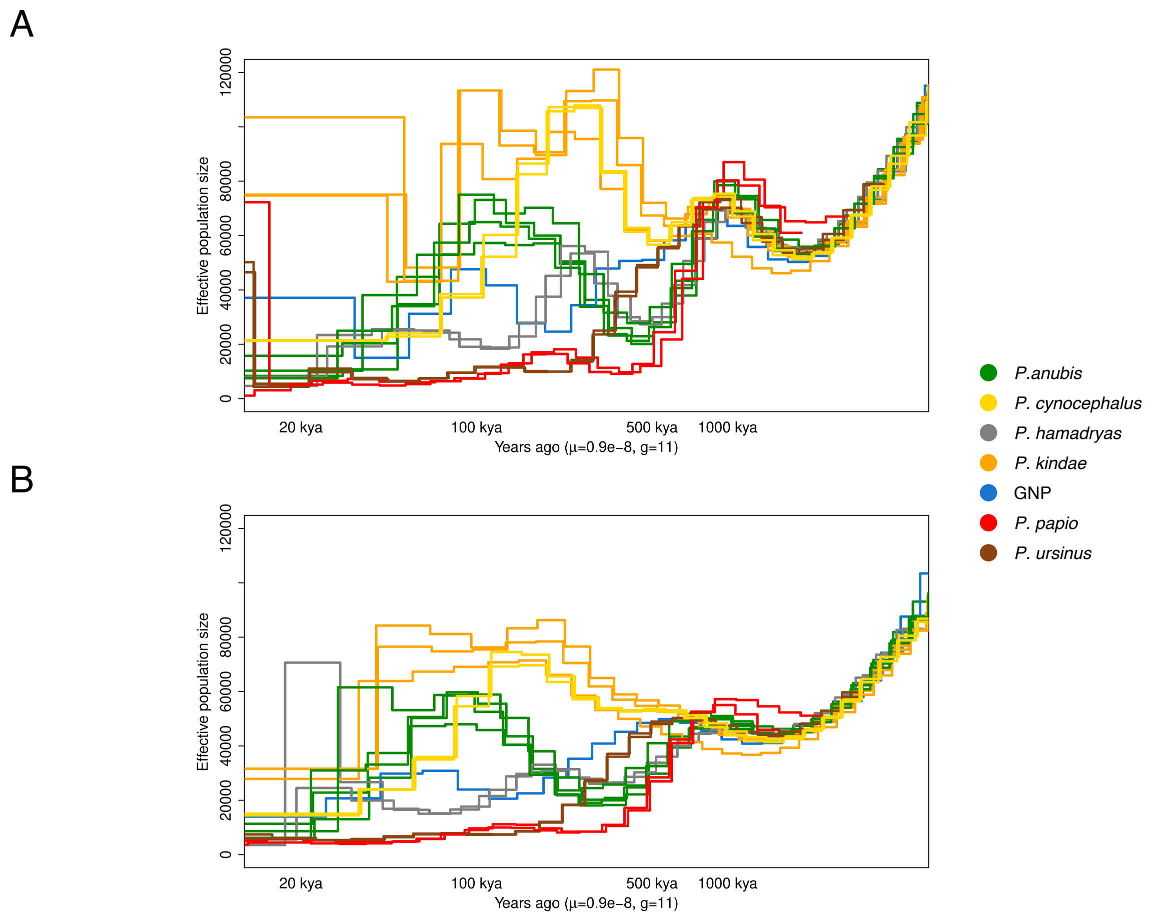

Figure S5. **Full PSMC results.** All *Papio* individuals are included, colored by species grouping, for autosomal chromosomes 1-20. (A) PSMC results including all sites. (B) PSMC results excluding repetitive regions as indicated by RepeatMasker.

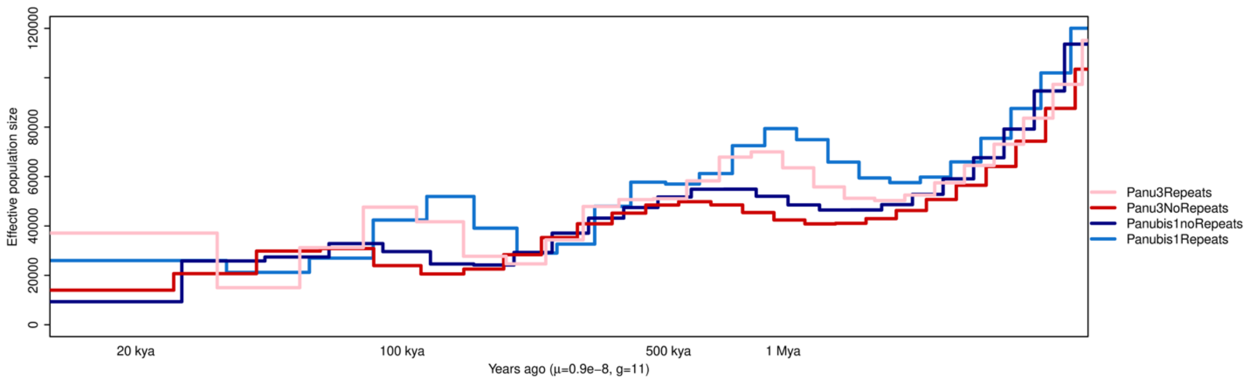

Figure S6. **Comparing assemblies Panu_3.0 and Panubis1.0 with PSMC.** GNP individually colored by results pertaining to whether RepeatMasker was used and which assembly the GNP individual was mapped against. PSMC results are for all autosomal chromosomes 1-20.

Characterizing P. cynocephalus ancestry in P. anubis

We validated our search for signatures of admixture in the GNP baboon by similarly testing one *P. anubis* sample previously reported to be the result of gene flow between *P. anubis* and *P. cynocephalus* (sample 30877; [1]. Significant f_3 values were obtained for individual 30877 when considered as sources on one side either *P. cynocephalus* or *P. ursinus* and on the other the rest of the *P. anubis* individuals. When other *P. anubis samples* were similarly tested none showed significant f_3 results (Table S6). Sample 30877 was further tested using the *D*-statistics in the form *D*(gelada, X, 30877, other *P. anubis* baboons). The sample 30877 showed strong deviations from zero when *P. cynocephalus* baboons were tested (Z = 12.78) (Figure 2a). A similar pattern, but weaker, was observed when other species evolutionary close to *P. cynocephalus* were tested (*P. ursinus* and *P kindae*). Conversely, the group comprising the remaining three *P. anubis* samples showed an increase in allele sharing with *P. papio* and *P. hamadryas* baboons.

Following the results of the f_3, f_4 and qpGraph analyses, we proceeded to estimate what fraction of genomic ancestry of sample 30877 which originated from *P. cynocephalus*. In doing so, we calculated the f_4 ratio alpha in the form f_4(gelada, cynocephalus, 30877, papio)/f_4(*gelada, cynocephalus; cynocephalus, papio*). The estimated value of alpha was 11%, lower than the 21% *P. cynocephalus* contribution previously reported using a different approach [1] (Table S7). We also estimated the f_4 ratio alpha considering only X chromosome SNPs. Estimates were positive (~4%) but not significant (Table S7).

Encouraged by the identification of clear signatures of admixture in *P. anubis* sample 30877, we decided to further characterize the presence of *P. cynocephalus* ancestry in this individual. We took advantage of the presence of multiple *P. anubis* genomes and used a local ancestry estimation

approach (ELAI) to assign genomic regions as having either a *P. cynocephalus or P. papio* origin (the latter being evolutionary close to *P. anubis*; [1]). A detailed recombination map for *Papio* is missing, so we considered a fixed recombination rate of 1cM/1Mb, and a generation time of 11 years, as in previous investigations [1, 3]. ELAI uses local haplotype features to characterize the defined source populations and assigns each admixed target haplotype to one of the sources based on the similarity of their features. Thus, ELAI describes the ancestry fragments inherited by the source populations along the target individuals' chromosomes. The ancestry deconvolution analyses on the *P. anubis* populations showed that only one individual (30877) had traces of both *P. cynocephalus* (11%) and *P. papio* (83%) ancestries, while the other *P. anubis* individuals (30977, L142, LIV5) had virtually all fragments deriving from a *P. papio*-like ancestry (Table S8). On the contrary, results of local ancestry analyses on sample GNP revealed that 81% of the fragments were assigned as having *P. ursinus* ancestry, < 1% to *P. cynocephalus* ancestry and 17% of the sequences could not be assigned to either putative source, despite the f_4 ratio suggested around 8% of the genome being shared with *P. cynocephalus*. Since the GNP results may be biased due to the small number of available samples (targets and sources), we tested the impact of using a single genome by running ELAI considering only the *P. anubis* sample known to be admixed (30877). The results on sample 30877 in this configuration were not consistent with the ELAI results obtained when the entire *P. anubis* population was considered, as only 7% of the sequences were assigned to the *P. cynocephalus* ancestry (Table S8). This discrepancy may be due to the inability of this approach to fully characterize the haplotype structure when sources and targets are represented by a small number of genomes. The failure in detecting *P. cynocephalus* ancestry in the GNP sample might be therefore similarly explained and we caution that local ancestry we performed on GNP may therefore be underpowered. Ancestry misassignment of GNP genomic

segments could be also driven by the antiquity of the shared haplotypes, with older haplotypes, smaller and more differentiated, being potentially more difficult to assign.

The fraction of sample 30877 genome identified as having *P. cynocephalus* baboon ancestry (Table S8) is similar to the contribution obtained using the f_4 ratio (Table S7). We then used the number of local ancestry switches from the fragments detected in sample 30877 to estimate the number of generations elapsed since the *P.anubis/P.cynocephalus* ancestors came into contact. The number of ancestry switches supported an event dated to ~40 generations ago (Figure S7), although the almost perfect mirroring of ancestry blocks along the two haploid genomes of the 30877 individual may point to a low phasing quality. Therefore, assuming most ancestry blocks to have been duplicated on both chromosomes due to poor phasing, one may caution that the 40 generations estimated above may be reduced up to 50% (20 generations since the admixture event). We independently validated these results by running ALDER and MALDER, which explore the distribution of linkage disequilibrium across the genome between pairs of alleles derived from different sources, assuming that all the analyzed recipient individuals share a similar history of admixture. Using *P. cynocephalus* and *P. hamadryas* as sources of the variation present in the whole *P. anubis* dataset (comprising four individuals) a significant signal was identified for an admixture event dating to 15 generations ago (Table S9). The younger estimate of ALDER can be reconciled with the one obtained by directly measuring the ancestry switches after the ancestry deconvolution, given that the former averages the admixture history of one admixed *P. anubis* (sample 30877) with other putatively unadmixed ones, while the latter is sample specific. In order to explore if additional more ancient events might have been present, we repeated the analysis using different bin sizes, which are supposed to capture signals at smaller distances. No additional admixture events were detected. The analysis conducted after removal of 30877 still led to the

identification of only a single admixture signal, 15 generations ago (Table S9). Our detection of a recent *P. cynocephalus* / *P. anubis* admixture event, between 15 and 20 generations ago, is consistent with the 21 generations previously reported using a different approach [1].

We evaluated the impact that this recent admixture event had on *P. anubis* variation. The heterozygosity estimated at individual level for *P. anubis* is in the middle of the *Papio* range, with sample 30877 showing approximately 20% more heterozygous positions than other *P. anubis* individuals (0.12 vs 0.10; Figure 1c). We identified the genomic regions in sample 30877 for which a *P. cynocephalus* ancestry was assigned using ELAI and estimated the heterozygosity when variation within these regions was not taken in consideration. When the same fragments were similarly excluded from all the *Papio* samples here analyzed, the overall pattern of diversity across samples remained similar, except for 30877 which showed a value of heterozygosity comparable to those estimated in the other *P. anubis* samples (~0.10; Table S2).

Table S6. *Please see Supplementary Tables spreadsheet, Table S6.* f_3 analyses.

Table S7. *Please see Supplementary Tables spreadsheet, Table S7.* f_4 ratio estimates.

Figure S7. ELAI karyotype ancestry per chromosome for admixed baboon 30877 using fixed recombination rate. *P. papio* tracts are colored in blue and *P. cynocephalus* tracts in pink. A and B indicate maternal and paternal haplotypes.

Figure S8. ELAI karyotype ancestry per chromosome for admixed baboon 30877 using variable recombination map. *P. papio* tracts are colored in blue and *P. cynocephalus* tracts in pink. A and B indicate maternal and paternal haplotypes.

Table S8. *Please see Supplementary Tables spreadsheet, Table S8.* Local Ancestry analyses (ELAI results).

Fig. S7

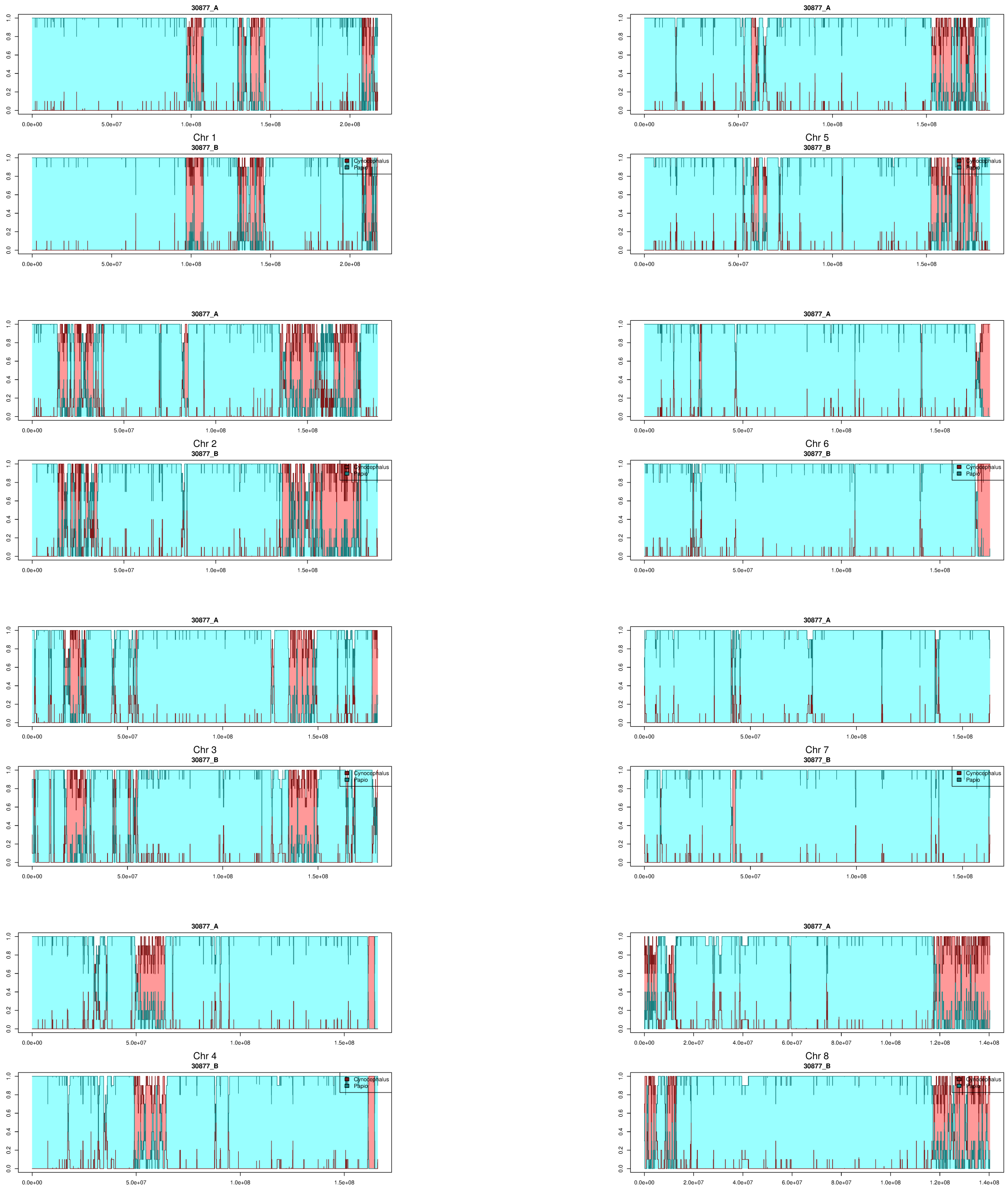
30877_A
Chr 1
30877_B
Cynocephalus
Papio
30877_A
Chr 5
30877_B
Cynocephalus
Papio
30877_A
Chr 2
30877_B
Cynocephalus
Papio
30877_A
Chr 6
30877_B
Cynocephalus
Papio
30877_A
Chr 3
30877_B
Cynocephalus
Papio
30877_A
Chr 7
30877_B
Cynocephalus
Papio
30877_A
Chr 4
30877_B
Cynocephalus
Papio
30877_A
Chr 8
30877_B
Cynocephalus
Papio

Fig. S7

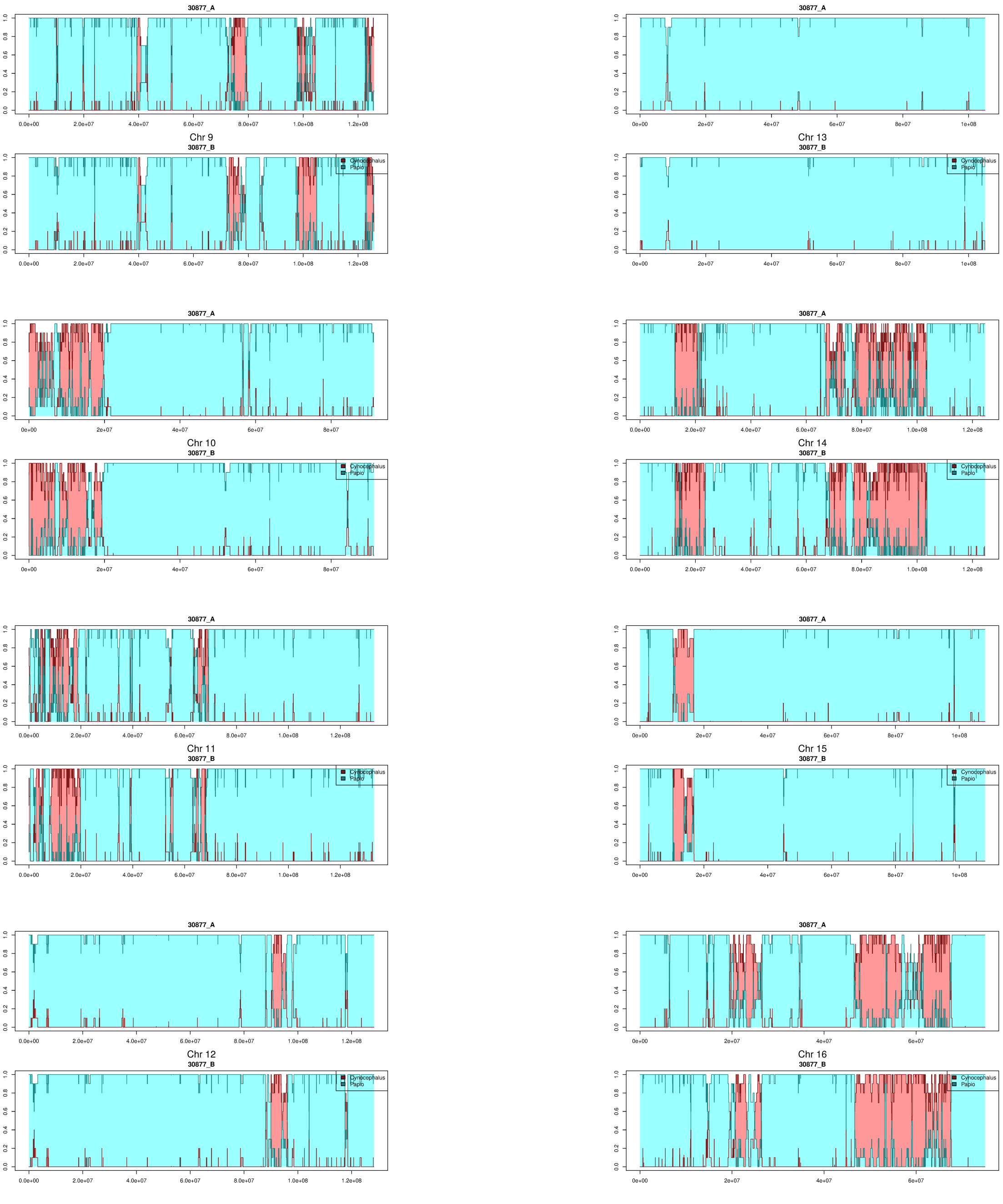

30877_A
Chr 9
30877_B
Cynocephalus
Papio
30877_A
Chr 13
30877_B
Cynocephalus
Papio
30877_A
Chr 10
30877_B
Cynocephalus
Papio
30877_A
Chr 14
30877_B
Cynocephalus
Papio
30877_A
Chr 11
30877_B
Cynocephalus
Papio
30877_A
Chr 15
30877_B
Cynocephalus
Papio
30877_A
Chr 12
30877_B
Cynocephalus
Papio
30877_A
Chr 16
30877_B
Cynocephalus
Papio

Fig. S7

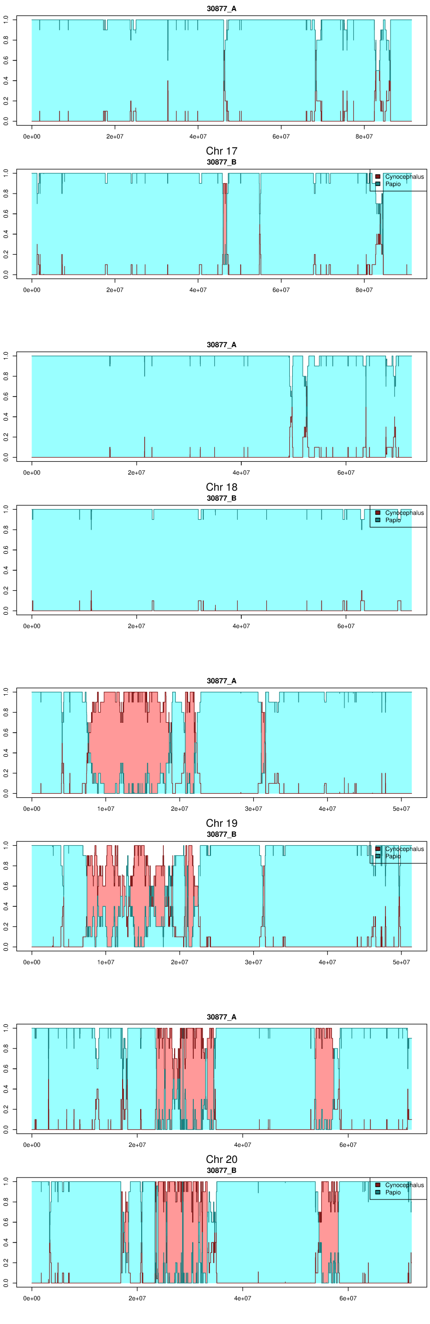
30877_A
Chr 17
30877_B
Cynocephalus
Papio
30877_A
Chr 18
30877_B
Cynocephalus
Papio
30877_A
Chr 19
30877_B
Cynocephalus
Papio
30877_A
Chr 20
30877_B
Cynocephalus
Papio

Fig. S8

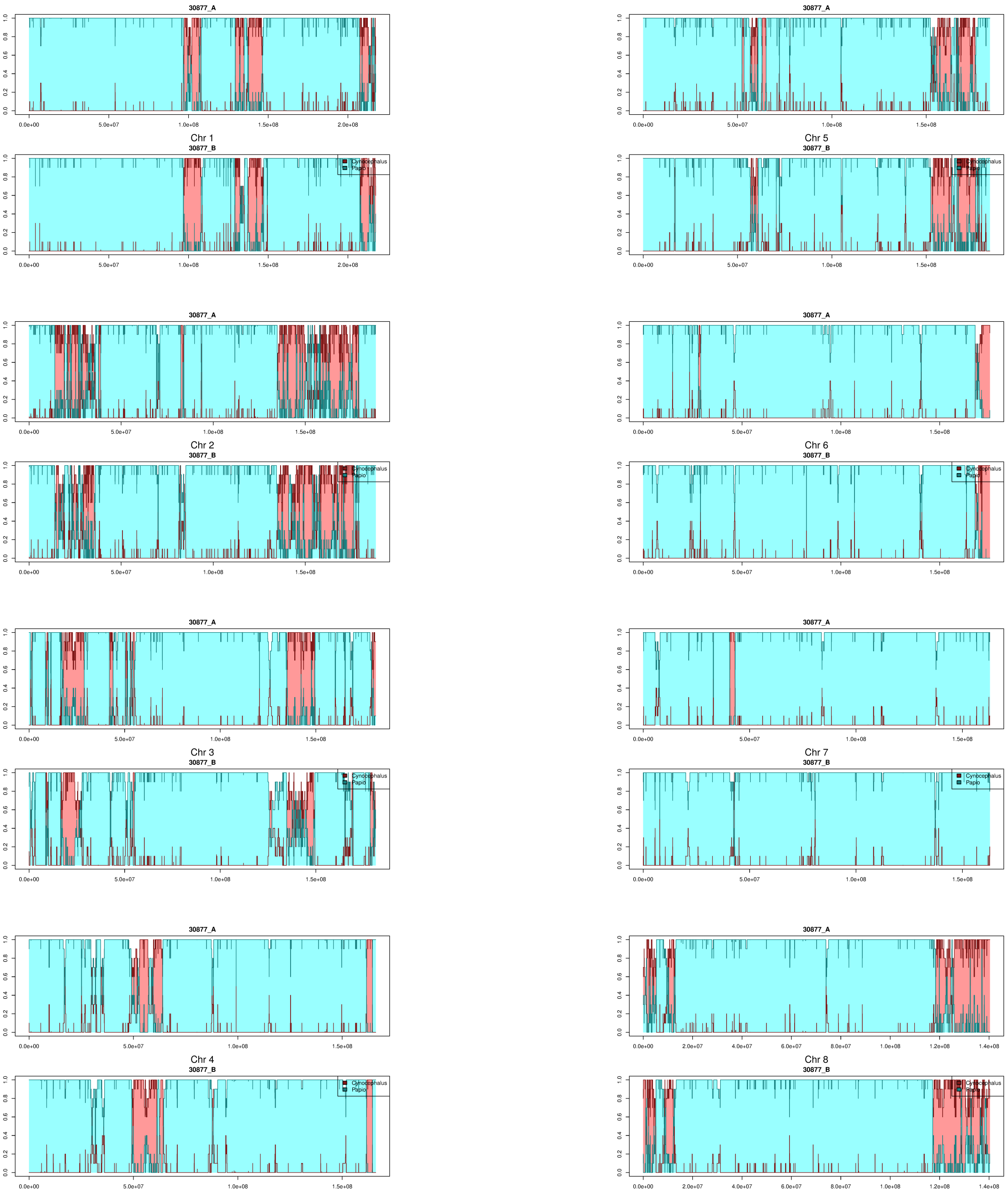

30877_A
30877_B
Chr 1
Chr 2
Chr 3
Chr 4
Chr 5
Chr 6
Chr 7
Chr 8
Cynocephalus
Papio

Fig. S8

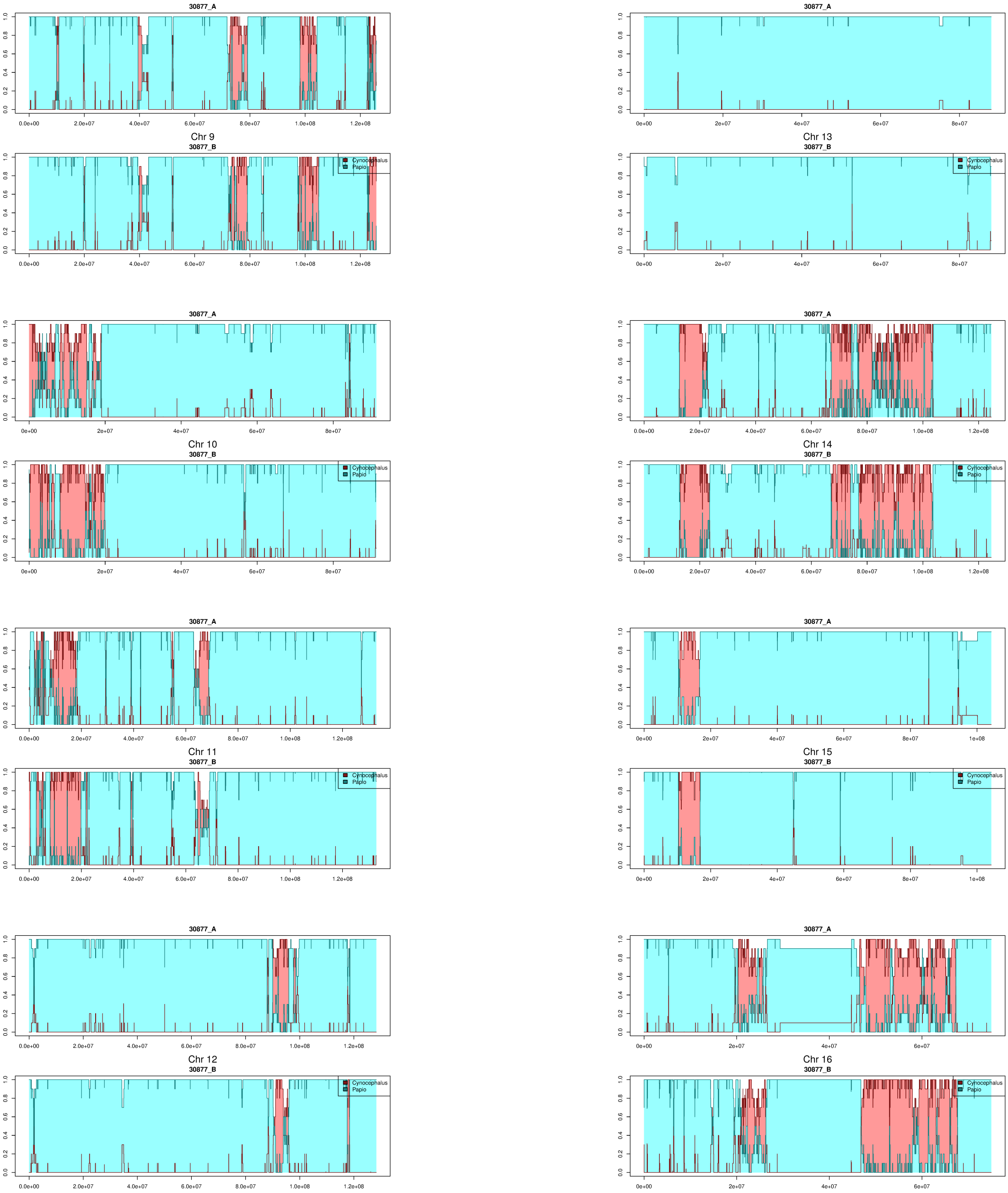
30877_A
Chr 9
30877_B
Cynocephalus
Papio
30877_A
Chr 13
30877_B
Cynocephalus
Papio
30877_A
Chr 10
30877_B
Cynocephalus
Papio
30877_A
Chr 14
30877_B
Cynocephalus
Papio
30877_A
Chr 11
30877_B
Cynocephalus
Papio
30877_A
Chr 15
30877_B
Cynocephalus
Papio
30877_A
Chr 12
30877_B
Cynocephalus
Papio
30877_A
Chr 16
30877_B
Cynocephalus
Papio

Fig. S8

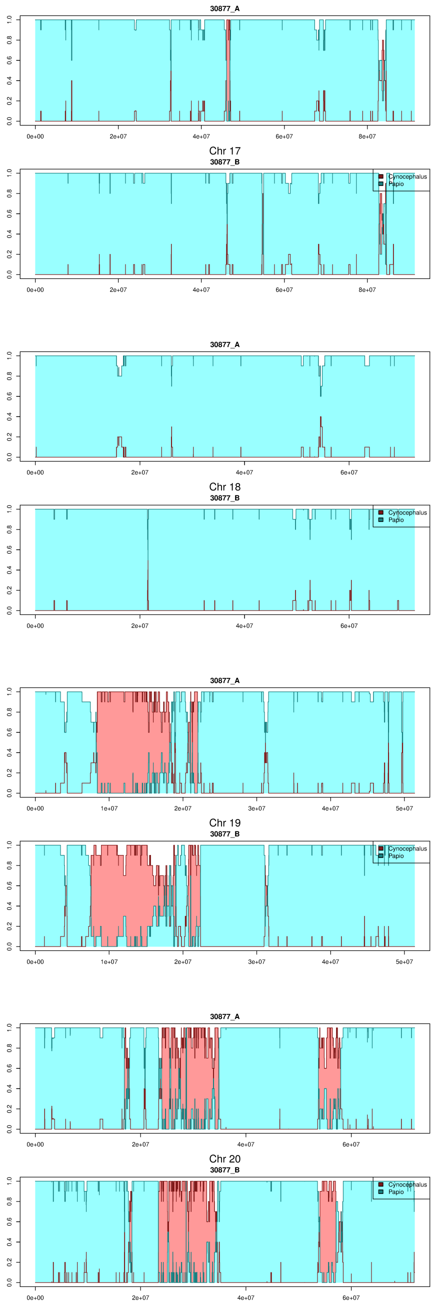
30877_A
Chr 17
30877_B
Cynocephalus
Papio
30877_A
Chr 18
30877_B
Cynocephalus
Papio
30877_A
Chr 19
30877_B
Cynocephalus
Papio
30877_A
Chr 20
30877_B
Cynocephalus
Papio

References

1. Rogers J, Raveendran M, Harris RA, Mailund T, Leppälä K, Athanasiadis G, et al. The comparative genomics and complex population history of baboons. Sci Adv. 2019;5:eaau6947.

2. Zinner D, Wertheimer J, Liedigk R, Groeneveld LF, Roos C. Baboon phylogeny as inferred from complete mitochondrial genomes. Am J Phys Anthropol. 2013;150:133–40.

3. Wall JD, Schlebusch SA, Alberts SC, Cox LA, Snyder-Mackler N, Nevonen KA, et al. Genomewide ancestry and divergence patterns from low-coverage sequencing data reveal a complex history of admixture in wild baboons. Mol Ecol. 2016;25:3469–83.

4. Mölder F, Jablonski KP, Letcher B, Hall MB, Tomkins-Tinch CH, Sochat V, et al. Sustainable data analysis with Snakemake. F1000Research. 2021;10:33.

5. Vasimuddin M, Misra S, Li H, Aluru S. Efficient Architecture-Aware Acceleration of BWA-MEM for Multicore Systems. 2019 IEEE International Parallel and Distributed Processing Symposium (IPDPS). 2019.

6. Li H, Handsaker B, Wysoker A, Fennell T, Ruan J, Homer N, et al. The Sequence Alignment/Map format and SAMtools. Bioinformatics. 2009;25:2078–9.

7. Broad Institute. Picard Tools 2.22.1. 2020. http://broadinstitute.github.io/picard/.

8. Korneliussen TS, Albrechtsen A, Nielsen R. ANGSD: Analysis of Next Generation Sequencing Data. BMC Bioinformatics. 2014;15:356.

9. Cortez D, Marin R, Toledo-Flores D, Froidevaux L, Liechti A, Waters PD, et al. Origins and functional evolution of Y chromosomes across mammals. Nature. 2014;508:488–93.

10. Coordinators NR, NCBI Resource Coordinators. Database resources of the National Center for Biotechnology Information. Nucleic Acids Research. 2012;41:D8–20.

11. Camacho C, Coulouris G, Avagyan V, Ma N, Papadopoulos J, Bealer K, et al. BLAST : architecture and applications. BMC Bioinformatics. 2009;10:421.

12. Kumar S, Stecher G, Li M, Knyaz C, Tamura K. MEGA X: Molecular Evolutionary Genetics Analysis across Computing Platforms. Mol Biol Evol. 2018;35:1547–9.

13. Castresana J. Selection of conserved blocks from multiple alignments for their use in phylogenetic analysis. Mol Biol Evol. 2000;17:540–52.
